# BrainAGE as a measure of maturation during early adolescence

**DOI:** 10.1162/imag_a_00037

**Published:** 2023-11-30

**Authors:** Lucy B. Whitmore, Sara J. Weston, Kathryn L. Mills

**Affiliations:** Department of Psychology, University of Oregon, Eugene, OR, United States; PROMENTA Research Center, Department of Psychology, University of Oslo, Oslo, Norway

**Keywords:** puberty, cognition, neurodevelopment, tween, longitudinal, brain age

## Abstract

The Brain-Age Gap Estimation (BrainAGE) is an important new tool that purports to evaluate brain maturity when used in adolescent populations. However, it is unclear whether BrainAGE tracks with other maturational metrics in adolescence. In the current study, we related BrainAGE to metrics of pubertal and cognitive development using both a previously validated model and a novel model trained specifically on an early adolescent population. The previously validated model was used to predict BrainAGE in two age bands, 9-11 and 10-13 years old, while the novel model was used with 9-11 year olds only. Across both models and age bands, an older BrainAGE was related to more advanced pubertal development. The relationship between BrainAGE and cognition was less clear, with conflicting relationships across the two models. Additionally, longitudinal analysis revealed moderate to high stability in BrainAGE across early adolescence. The results of the current study provide initial evidence that BrainAGE tracks with some metrics of maturation, including pubertal development. However, the conflicting results between BrainAGE and cognition lead us to question the utility of these models for non-biological processes.

## 1 Introduction

Over the last 25 years, we have learned a considerable amount about the prolonged development of the human brain, due in part to advances in neuroimaging technologies and methods (Bethlehem et al., 2022; Mills et al., 2016; Raznahan et al., 2011; Wierenga et al., 2014). Brain development progresses differently across individuals (Mills et al., 2021), with different patterns and trajectories theorized to relate to outcomes such as psychopathology (Shaw et al., 2010). While longitudinal data are necessary to track individual trajectories, most neuroimaging studies that examine the brain across the lifespan are cross-sectional, and there has been an increasing desire to find ways to measure individual differences in brain maturation using methods that are compatible with cross-sectional data. The Brain-Age Gap Estimation (BrainAGE) has been proposed as one approach to assess the maturity of an individual’s brain, as this measure reflects the difference between an individual’s chronological age and their age as predicted by machine-learning algorithms trained on neuroimaging data, often structural MRI (Brown et al., 2012; Franke et al., 2010). With this approach, an individual receives an estimated brain age that can differ from their chronological age.

### 1.1 BrainAGE as a measure of aging and pathology

BrainAGE has most frequently been used in older adult populations, as it was originally proposed as a tool to research and assess risk for aging-related diseases (Franke et al., 2010). For older adults, differences between an individual’s estimated brain age and chronological age have been thought to reflect potential deviations from a normative aging trajectory related to various health concerns. This has been supported by previous work that has demonstrated having a BrainAGE that is older than one’s chronological age is related to metrics and outcomes of cognitive aging and decline, such as Alzheimer’s, in older adults (Biondo et al., 2022; Franke & Gaser, 2012, 2019). Additionally, across adulthood, having an older BrainAGE than one’s chronological age has also been associated with psychopathologies, including depression and schizophrenia (Han et al., 2021, 2022; Schnack et al., 2016).

### 1.2 BrainAGE in adolescence

Although BrainAGE is more commonly used in investigations of older adult populations, researchers are starting to use BrainAGE as a tool to advance current theories in developmental cognitive neuroscience. As BrainAGE was not originally developed as a measure of individual differences in adolescent brain maturation, the implications for interpreting BrainAGE are less clear in this population than in older populations where an older-appearing brain is more straightforwardly related to aging-related diseases. Conventionally, differences between BrainAGE and chronological age have been interpreted as reflecting accelerated or decelerated brain maturation in children and adolescents, though this has not been validated with longitudinal data (Franke et al., 2012; Lewis et al., 2018).

BrainAGE prediction based on structural MRI has been shown to accurately predict age in adolescents as measured by mean absolute errors in the 1- to 2-year range (Franke et al., 2012). Since the inception of using BrainAGE in adolescent populations, there have been efforts to link an adolescent’s BrainAGE to psychopathologies, which often emerge during this time. Recently, BrainAGE has shown promise as a potential biomarker of mental health issues in adolescent populations. Notably, having a BrainAGE that is older than one’s chronological age has been linked to major depression, functional impairment, and risk of psychosis (Chung et al., 2018; Cropley et al., 2021; Drobinin et al., 2022). This is consistent with the idea that deviations from typical developmental trajectories play a role in the development of psychological disorders, as hypothesized by Shaw et al. (2010). Similarly, the Stress Acceleration Hypothesis (Callaghan & Tottenham, 2016) proposes that early life stress accelerates neural development, potentially resulting in short-term benefits but long-term vulnerability to psychopathology.

However, in recent years, BrainAGE has also been theorized to reflect more than just accelerated or decelerated brain maturation (Ball et al., 2021, Kelly et al., 2022; Vidal-Pineiro et al., 2021). In adults, BrainAGE has been found to relate to birth weight and polygenic brain age scores (Videl-Pineiro et al., 2021). Additionally, adult BrainAGE varies over the course of the menstrual cycle (Franke et al., 2015). In children and adolescents, BrainAGE has been related to genetic factors (Brouwer et al., 2021). Despite this, the success of BrainAGE prediction in adolescent populations suggests its potential use as a clinically relevant biomarker; however, given a lack of clarity around interpretations of adolescent BrainAGE, additional validation is needed before BrainAGE can be recommended for clinical and applied settings. In particular, it remains unclear as to which aspects of maturation are captured by this measure and which are not. A challenge to validation is the lack of conceptual clarity around the concept of “brain maturity” and what it means for a brain to be mature (Somerville, 2016).

The validation of any measure interpreted as reflecting a maturational process is an important step, and one that has been accomplished for developmental metrics such as the Tanner stages, also known as the Tanner Scale (Tanner, 1962). The Tanner stages, developed in the 1960s, describe and quantify physical changes associated with puberty. The stages were validated by observing adolescents and correlating physical changes with the corresponding stage, making it a widely accepted measure of physical maturation in clinical and research settings. In the case of adolescent BrainAGE, we would expect a similar relationship to be present, where BrainAGE is related to other maturational metrics that develop over adolescence. In the current study, we explore the relationship of BrainAGE to two domains of changes that are occurring in this time: pubertal and cognitive development.

### 1.3 Brain maturation and puberty

Pubertal development is often measured in stages, such as Tanner stages or using the Pubertal Development Scale (PDS) (Petersen et al., 1988; Tanner, 1962). Tanner stages measure physical development, based on changes in pubic hair, genitalia, and breast growth (for females). Based on these metrics, Tanner Stage 1 represents pre-puberty, Stages 2-4 represent mid/intermediate puberty, and Stage 5 represents reproductive maturity. The Pubertal Development Scale examines similar, though not entirely overlapping, measures as Tanner stages and includes questions about characteristics such as body hair, growth in height, and other secondary sex characteristics (Petersen et al., 1988). Pubertal hormones such as testosterone or estradiol are also commonly examined in relation to pubertal development, but no current scale exists to assess pubertal development progress based on levels of certain hormones.

Independent of age, pubertal stage has been related to changes in brain structures, both cortical and subcortical. While cross-sectional relationships between subcortical volumes and pubertal development have found mixed results, longitudinal work has more consistently found a positive relationship between pubertal stage and amygdala and hippocampus volumes and a negative relationship between pubertal stage and nucleus accumbens, caudate, putamen, and globus pallidus volumes (Goddings et al., 2014; Herting and Sowell, 2017). Pubertal stage has also been found to be a better predictor of development than age in a number of subcortical regions, including the caudate, pallidum, and hippocampus (Wierenga et al., 2018).

A previous literature review found that both cross-sectional and longitudinal studies report widespread patterns of reductions in cortical gray matter in relation to pubertal development, both stage and timing (Vijayakumar et al., 2018). Notably, significant negative associations have been found between the pubertal hormone testosterone and cortical thinning in regions, including the left posterior cingulate, precuneus, dlPFC, and ACC in males and right somatosensory cortex in females (Nguyen et al., 2013). Additionally, change in Tanner stage has been related to rates of change in cortical surface area, where a main effect of stage was found for decreases in left precuneus surface area (Herting et al., 2015). More recent work has shown relationships between pubertal stage and increased cortical thinning in frontal and parietal cortices, as well as some temporal regions, independent of age (Vijayakumar et al., 2021).

In terms of pubertal development, recent work exists on the relationship between puberty and adolescent BrainAGE. Using a convolutional neural net-based BrainAGE model trained on a lifespan population (5-93 years old), Holm et al. (2023) found a positive relationship between BrainAGE and parent-report pubertal development in a sample of 9-13 year olds from the ABCD study. Additionally, Holm and colleagues found a small association between the annualized rate of change in PDS scores and the annualized rate of change in BrainAGE, controlling for the annualized rate of change in chronological age.

Additionally, Dehestani et al. (2023) found that earlier pubertal timing was associated with an older BrainAGE in a sample of 9-13 year old participants from the ABCD study. Pubertal timing was measured using “pubertal age,” a new measure that uses observed physical development (parent-report PDS) and pubertal hormones (testosterone and DHEA) to predict pubertal timing using a framework similar to that used for BrainAGE (Dehestani et al., 2023). BrainAGE was predicted from 90 features of cortical volume, surface area, and thickness, as well as subcortical volume, using a support vector regression framework. The BrainAGE model was trained and tested on a sample of 9-13 year olds from the ABCD Study.

Both of the previous studies examined pubertal development in the ABCD Study cohort, but each captured unique aspects of the relationship between BrainAGE and puberty. Notably, the Holm analyses used a CNN-based lifespan model to examine pubertal stage, while the Dehestani model used was trained on explicit measurements of thickness, volume, and area and examined in relation to pubertal timing.

### 1.4 Brain maturation and cognition

In addition to pubertal changes, adolescents undergo a number of cognitive changes, such as improved working memory performance and inhibitory control (Cromer et al., 2015; Davidson et al., 2006). Performance on the NIH Toolbox Cognition Battery, a commonly used measure of cognition, has been shown to improve cross-sectionally and longitudinally over early-to-mid adolescence (Anokhin et al., 2022). Additionally, working memory improvements in adolescence have been found to be associated with cortical volume reductions in bilateral prefrontal and posterior parietal regions and in regions around the central sulci (Tamnes et al., 2013).

### 1.5 BrainAGE and cognition in adolescence

Past research on cognition and BrainAGE in adolescence has found mixed results, with some work indicating that a positive BrainAGE is related to faster processing speed (Erus et al., 2015) and others finding that a negative BrainAGE was associated with better cognitive performance (Lewis et al., 2018). Additionally, some studies have found very small or non-significant effects (Ball et al., 2017, 2021; Kelly et al., 2022; Khundrakpam et al., 2015).

These studies exhibit a multitude of sources of variation, spanning from model features employed to train BrainAGE models, to participant age ranges, as well as the types of cognitive measures employed. In the studies described above, model features employed encompassed cortical thickness exclusively, T1 white/gray contrast, cortical thickness/volume/area, and subcortical volume, as well as the combination of gray matter, white matter, and ventricular measures. Parcellation methods varied significantly as well. Cognitive measurements employed in the studies were also highly varied, including IQ, measures of speed and accuracy on the Penn Computerized Neurocognitive Battery, and the NIH Toolbox Cognition Battery (Gershon et al., 2013; Gur et al., 2012). Although all of the studies incorporated adolescent participants, the age ranges varied significantly and the distribution of cognitive data across the age range was not always clearly indicated. Age ranges in certain studies were as broad as 8-22, 3-21, and 4.5-18.5 years, while Kelly et al. (2022) was restricted to cognition scores from participants who were 13 years old.

## 2 Current Study

In the current study, we examine the relationship between BrainAGE and metrics of pubertal and cognitive maturation within two narrow age bands in early adolescence, 9-11 years and 10-13 years. For BrainAGE models trained on structural MRI data, such as the models in this paper, we expect that BrainAGE would track with maturational metrics that are reflected in changes to brain structure and change during these age ranges, including pubertal and cognitive development. Additionally, we examine the stability of BrainAGE in early adolescence in a longitudinal analysis.

The current study used three different BrainAGE models in order to provide additional confidence in our results by comparing models trained on both wide and narrow age ranges in order to balance specificity and replication. This strategy enabled us to balance providing the models with extensive data from our age range of interest, while not overly restricting our training data and possible predictions. In Study 1, we used an existing, previously validated BrainAGE model trained on a wide age range to predict BrainAGE in a sample of early adolescents (Drobinin et al., 2022).

In Study 2, we trained and tested two new BrainAGE models trained specifically on our age ranges of interest (9-11 years old and 10-13 years old). The development of BrainAGE models trained specifically on data from early adolescents is novel, and their usage reflects the unique structural changes occurring during this age range. The goal of the novel models is to capture the specific changes happening in these ranges, as opposed to the dynamic changes occurring over all of adolescence. While BrainAGE models exist that include adolescents, none sample early adolescence specifically, despite this being a unique time for structural brain development. The novel models are available for public use and can be found at https://github.com/LucyWhitmore/BrainAGE-Maturation.

Using predictions from all models, BrainAGE estimates were examined in association with youth-report pubertal development, parent-report pubertal development, and cognition scores.

As the goal of the study was to examine relationships between BrainAGE and other forms of maturation and determine whether these relationships replicate across models and age bands, the methods of Study 1 and Study 2 were kept as similar as possible. In particular, model training code, analysis procedures, and pubertal/cognitive measures were identical across Study 1 and Study 2. Differences in samples and procedures are described where present. In the following sections, we discuss the studies separately, first detailing the procedures and results of Study 1, then the procedures and results of Study 2. Following Studies 1 and 2, we present longitudinal analyses of BrainAGE stability across both studies. Finally, we jointly discuss the implications, limitations, and future directions of the Studies 1 and 2.

### 2.1 Study 1

#### 2.1.1 Participants

##### 2.1.1.1 Model training

The model used in Study 1 was a previously validated BrainAGE model, created for use in Drobinin et al. (2022). The model was trained on 1,299 participants aged 9-19 years old, from a composite of six samples. Participants were members of the Autism Brain Imaging Data Exchange (ABIDE) Child Mind Institute: Healthy Brain Network (CMI), Consortium for Reliability and Validity, NIH MRI Study of Normal Brain Development, and Pediatric Imaging, Neurocognition, and Genetics (PING) cohorts. Distributions of age and gender per sample are available in Drobinin et al. (2022). Inclusion criteria included being within the age range of 9-19 years old and passing automated MRI quality control procedures. Additionally, only participants without psychiatric disorders and with an IQ over 75 were included in model training.

##### 2.1.1.2 Model prediction and analyses

Adolescents included in the model predictions and analyses were participants in the Adolescent Brain Cognitive Development (ABCD) Study. The ABCD Study is a large longitudinal study that has recruited participants from 21 sites across the United States. Informed consent was obtained from all participants and their parents. For further details on recruitment procedures in the ABCD Study, see Garavan et al. (2018).

While the ABCD Annual Release 4.0 included both baseline data and data from the 1- and 2-year follow-ups, only baseline and the 2-year follow-up included imaging data. Baseline imaging data were available for 11,878 participants between the ages of 9-11 years old. After excluding participants with missing or low-quality structural MRI data, 11,402 participants remained (*M* = 9.92 years old, *SD* = 0.63 years). Follow-up imaging data were available for 7,827 participants between the ages of 10 and 13. After excluding participants with missing or low-quality structural MRI data, 7,696 participants remained (*M* = 11.94 years old, *SD* = 0.65 years). Additional participant demographic details are available in Table 1.

**Table 1.** Participant demographics (ABCD).

		Baseline (model 1)	Follow-up (model 1)	Baseline (model 2)	Follow-up (model 2)
Sex	Female	48% (*N*=5471)	46.3% (*N* = 3566)	48.4% (*N* = 2759)	47.3% (*N=* 1747)
	Male	52% (*N* = 5931)	53.7% (*N* = 4129)	51.6% (*N* = 2943)	52.7% (*N=* 1946)
Race/Ethnicity	Non-Hispanic White	53.3% (*N* = 5962)	55.1% (*N* = 4242)	51.6% (*N* = 2946)	55.4% (*N* = 2045)
	Hispanic/Latino	20.5% (*N* = 2332)	19.3% (*N* = 1487)	21.5% (*N* = 1223)	19.7% (*N* = 729)
	Non-Hispanic Black	15.4% (*N* = 1754)	13.1% (*N* = 1010)	14.3% (*N* = 815)	12.9% (*N*=476)
	Asian	2.1% (*N* = 244)	1.9% (*N* = 145)	2.1% (*N* = 122)	1.9% (*N* = 70)
	Native American/ Alaska Native	0.5% (*N* = 56)	0.6% (*N* = 44)	0.5% (*N* = 30)	0.5% (*N* = 20)
	Multiracial	12% (*N* = 1,370)	12.1% (*N* = 931)	12.1% (*N* = 688)	11.3% (*N* = 419)
	Additional race	4.6% (*N* = 528)	4.3% (*N* = 335)	4.8% (*N* = 277)	4.5% (*N* = 167)
Household income	<$5000	3.4 % (*N* = 392)	2.5% (*N* = 196)	3.3% (*N* = 188)	2.6 % (*N* = 96)
	$5,000-$11,999	3.5% (*N* = 398)	2.7% (*N* = 211)	3.5% (*N* = 199)	3% (*N* = 111)
	$12,000-$15,999	2.3% (*N* = 265)	1.8% (*N* = 140)	2.3% (*N* = 130)	1.9% (*N* = 72)
	$16,000-$24,999	4.4% (*N* = 497)	3.2% (*N* = 246)	4.2% (*N* = 238)	4% (*N* = 148)
	$25,000-$34,999	5.6% (*N* = 633)	5.2% (*N* = 402)	5.7% (*N* = 324)	5.6% (*N* = 208)
	$35,000-$49,999	7.8% (*N* = 889)	6.8% (*N* = 524)	8.3% (*N* = 471)	8.4% (*N* = 309)
	$50,000-$74,999	12.5% (*N* = 1,429)	12.7% (*N* = 980)	13.1% (*N* = 747)	14.3% (*N* = 528)
	$75,000-$99,999	13.3% (*N* = 1,520)	13.3% (*N* = 1,026)	12.8% (*N* = 725)	13.6% (*N* = 503)
	$100,000-$199,999	28.1 % (*N* = 3,201)	31.4% (*N* = 2,413)	27.6% (*N* = 1,575)	28.9% (*N* = 1067)
	≥ $200,000	10.6% (*N* = 1,208)	12.49% (*N* = 961)	10.6% (*N* = 603)	9.9% (*N* = 367)
	Missing	8.5% (*N* = 970)	7.7% (*N* = 596)	8.8% (*N* = 501)	7.7% (*N* = 284)
Model training & testing	# Participants (Training)	1,621	1,621	5,701	3,739
	Age range (training)	9-19 years, m = 13.5 ± 3.04	9-19 years, m = 13.5 ± 3.04	8.9-11.08 years, m = 9.92 ± 0.63	10.58-13.67years, m = 11.95 ± 0.66
	# Participants (testing)	11,402	7,695	5,701	3,693
	Age range (testing)	8.9-11.08 years, m = 9.92 ± 0.63	10.58-13.83 years, m = 11.94 ± 0.65	8.9-11.08 years, m = 9.92 ± 0.63	10.58-13.83 years, m = 11.94 ± 0.64

#### 2.1.2 Materials & methods

##### 2.1.2.1 MRI acquisition

For details on the acquisition of MRI images used to train the model used in Study 1, see Drobinin et al. (2022). For participants included in the analyses, detailed imaging procedures can be found in Casey et al. (2018) and Hagler et al. (2019). Harmonized protocols were used across 21 sites to collect imaging data using one of three 3 T scanner platforms (Phillips, Siemens, or GE) and a 32-channel head coil; 3-dimensional T1-weighted scans were collected with 1-mm voxel resolution.

##### 2.1.2.2 Model training

As described in Drobinin et al., 2022, model training was conducted within the tidymodels (version 0.1.1) framework, using the XGBoost ML algorithm (version 1.0.0.2). Scan age was predicted from 189 features, including cortical and subcortical volume and area measurements.

##### 2.1.2.3 MRI processing and model features

Model features included cortical gray matter volume and surface area measurements, as well as bilateral global and subcortical volume measurements. Cortical features were obtained using the Freesurfer default Desikan-Killiany atlas, while bilateral global and subcortical volume measures were obtained using the Freesurfer output. Data from the ABCD Study used for model testing were harmonized across sites using the longCombat package (Beer et al., 2020).

The longCombat package was used to account for the multisite structure and longitudinal nature of the ABCD Study.

The Drobinin et al. (2022) model was originally trained on 189 structural features, but only 175 of these features were available in the ABCD dataset. Therefore, for the current study, the model was used to predict BrainAGE based on the 175 available features (Supplementary Table 1).

##### 2.1.2.4 BrainAGE prediction

For the baseline sample, the Drobinin et al. (2022) model was used to predict BrainAGE for 11,402 adolescents (9-11 years old). For the 2-year follow-up sample, the model was used to predict BrainAGE for 7,696 adolescents (10-13 years old).

##### 2.1.2.5 Bias correction

Like other machine-learning models, BrainAGE models can be susceptible to prediction bias towards the group mean (Le et al., 2018). In the case of BrainAGE models, this bias often takes the form of younger participants being more likely to be predicted as slightly older than their chronological age, while older participants are predicted to be slightly younger. To adjust for this age bias, we followed the bias correction procedures described in Smith et al. (2019). Briefly, the bias correction procedures require fitting a linear model to the validation set and extracting the intercept and slope. To obtain a corrected estimate, the intercept is subtracted from predicted age, and then value is then divided by the slope. The slope and intercept are generalizable to new data, allowing these coefficients to be used to correct test set bias (Peng et al., 2021). Additionally, age was included as a covariate in analyses to further correct for age-related bias.

#### 2.1.3 Behavioral & survey measures

##### 2.1.3.1 Youth & parent report pubertal development

Both participants and their parents completed the Pubertal Development Scale (PDS; Barch et al., 2018; Petersen et al., 1988). The PDS is a five-item scale that measures pubertal stage. Of the five items, three are asked of every participant (related to growth in height, skin changes, and body hair growth), and two are dependent on the participants’ assigned sex at birth. The female version asks about the onset of menstruation and breast growth, while the male version asks about facial hair and vocal changes.

For each participant, mean scores were calculated for each of their self-report and parent-report scores (α = .53-.81, see Supplementary Table 2 for reliability estimates of the measures). Participants with incomplete data were excluded from the corresponding analyses.

##### 2.1.3.2 Cognition

Cognition was measured using the NIH Toolbox Cognition Battery (Bleck et al., 2013; Gershon et al., 2013; Hodes et al., 2013). The Toolbox uses seven cognitive tasks, which measure domains such as cognitive control, working memory, set shifting, and reading ability (Table 2).

**Table 2.** NIH Toolbox cognition tasks.

NIH Toolbox Flanker®	Cognitive Control/Attention
NIH Toolbox List Sorting Working Memory Test®	Working Memory; Categorization; Information Processing
NIH Toolbox Dimensional Change Card Sort®	Flexible thinking; concept formation; set shifting
NIH Toolbox Oral Reading Recognition Test®	Reading Ability; Language; Academic Achievement
NIH Toolbox Pattern Comparison Processing Speed®	Processing Speed; Information Processing
NIH Toolbox Picture Sequence Memory Test®	Visuospatial sequencing & memory
NIH Toolbox Picture Vocabulary Test®	Language; Verbal intellect

NIH Toolbox cognition tasks and their respective cognitive domains.

Summary scores were provided in the ABCD 4.0 data release for each participant based on their performance on the seven tasks, listed in Table 1, and described in detail in Luciana et al. (2018). Summary scores are presented as the participants’ performance compared to those in the NIH Toolbox normative sample, which includes nationally representative data from participants who are between 3-85 years old. Complete cognition summary scores were only available at baseline. Distributions of all maturational metrics are available in Table 3.

**Table 3.** Descriptive statistics.

	BrainAGE-corrected		Youth-report puberty		Parent-report puberty		Cognition	
Study	Mean ± SD	Range	Mean ± SD	Range	Mean ± SD	Range	Mean ± SD	Range
Study 1, baseline	0.68 ± 1.75	-4.97-8.52	1.71 ± 0.47	1.0-4.0	1.60 ± 0.48	1.0-4.0	86.34 ± 9.10	44-117
Study 1, follow-up	0.19 ± 1.98	-5.73-7.90	2.07 ± 0.65	1.0-4.0	2.12 ± 0.70	1.0-4.0	—	—
Study 2, baseline	-0.05 ± 2.19	-7.71-7.36	1.72 ± 0.48	1.0-4.0	1.60 ± 0.48	1.0-4.0	86.31 ± 9.15	44-115
Study 2, follow-up	0.01 ± 2.80	-9.68-9.84	2.08 ± 0.65	1.0-4.0	2.12 ± 0.71	1.0-4.0	—	—

#### 2.1.4 Statistical analyses

Statistical analyses were performed in RStudio (R version 4.1.1, RStudio version 2021.09.0). Associations with maturational metrics were analyzed with linear models, using the *lm()* function. Separate models were created for the relationship between BrainAGE and each maturational metric, where BrainAGE was regressed on each metric of puberty and cognition. Age was included as a covariate to reduce age-related bias in BrainAGE estimates. We report unstandardized effects and standard errors in-text and in Table 4. Standardized effects are available in Table 5. Full regression models are available in Supplementary Tables 3-12.

**Table 4.** Summary of results, unstandardized.

	Youth-report puberty			Parent-report puberty			Cognition		
	Beta	SE	p	beta	SE	p	beta	SE	p
Study 1, baseline	0.25	0.05	<.001	0.24	0.04	<.001	-0.003	0.002	<.001
Study 1, follow-up	0.50	0.04	<.001	0.58	0.03	<.001	—	—	—
Study 2, baseline	0.31	0.09	<.001	0.19	0.06	<.001	0.002	0.003	.005
Study 2, follow-up	0.39	0.08	<.001	0.42	0.07	<.001	-	-	-

**Table 5.** Summary of results, standardized.

	Youth-report puberty		Parent-report puberty		Cognition	
	beta	95% CI_	beta	95% CI_	beta	95% CI_
Study 1, baseline	0.07	[0.04, 0.09]	0.07	[0.05, 0.09]	-0.02	[-0.04, 0.0]
Study 1, follow-up	0.16	[0.14, 0.19]	0.21	[0.18, 0.23]	—	—
Study 2, baseline	0.07	[0.03, 0.10]	0.04	[0.01, 0.07]	0.01	[-0.02, 0.04]
Study 2, follow-up	0.09	[0.06, 0.13]	0.11	[0.07, 0.14]	—	—

#### 2.1.5 Results

##### 2.1.5.1 BrainAGE model performance

**Fig. 1.**
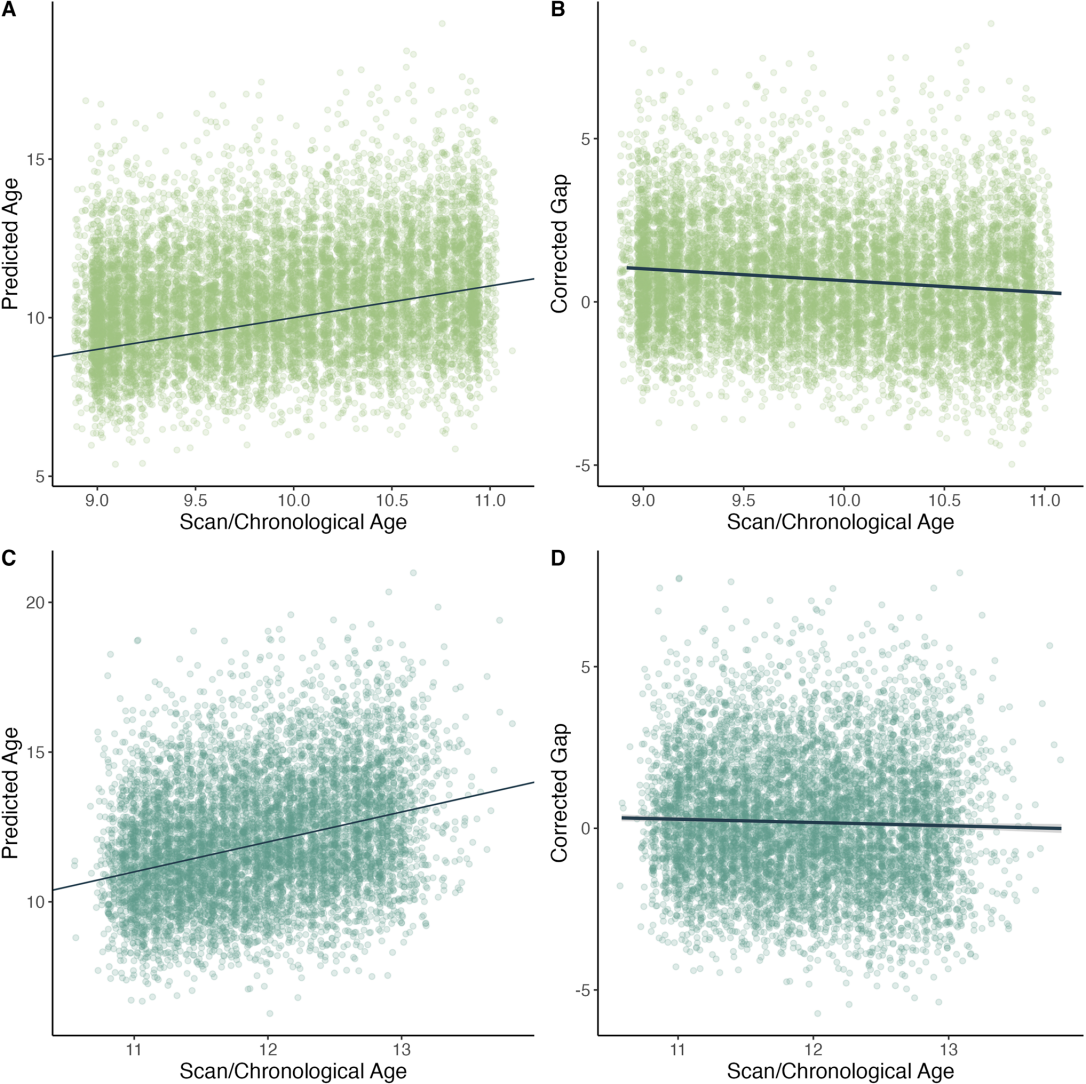
Age versus model predictions for the previously validated model. (A) Scan/chronological age versus model-predicted age for the baseline sample. The line represents an exact relationship between chronological age and predicted age. (B) Scan/chronological age versus the age-corrected brain-age gap, for the baseline sample. The regression line shows a slight age-related bias, with younger participants predicted to be slightly older than their chronological age. (C) Similar to (A), scan/chronological age versus predicted age for the follow-up sample. (D) Similar to (B) scan/chronological age versus the age-corrected brain-age gap for the follow-up sample. Again, the regression line shows a slight amount of age-bias, but less than at baseline.

In the original validation of the model, the model performed with a corrected mean absolute error (MAE) of 1.98 (Drobinin et al., 2022). This error is in line with prior adolescent BrainAGE models, which have performed with MAEs frequently in the 1-2 year range (Franke & Gaser, 2019). The existing model performed with a MAE of 2.32 before correction for age bias, and a corrected MAE of 1.45 on the ABCD data (baseline) and an uncorrected MAE of 1.3 (corrected MAE 1.58) at follow-up. Model performance is shown in Figure 1. A slight age bias was observed, as is common in these types of models, and was discussed previously. BrainAGE (predicted age subtracted from chronological age) had a correlation of -0.1 with chronological age at baseline, and -0.01 at follow-up.

##### 2.1.5.2 BrainAGE and maturational metrics

**Fig. 2.**
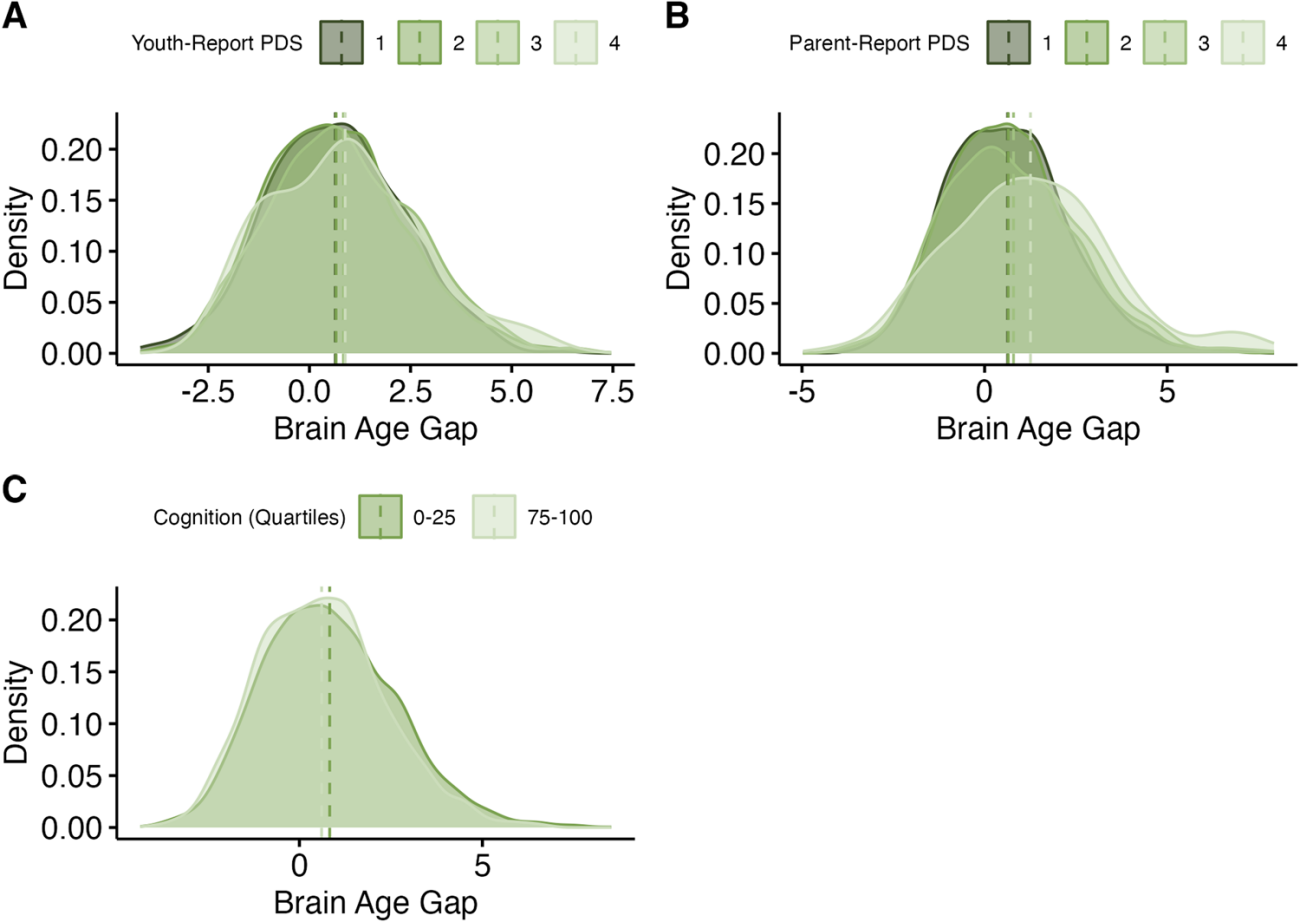
Results: Previously validated model, baseline sample. (A) Distributions of brain-age gaps, grouped by pubertal stage (youth-report). Means for each group are represented with vertical lines. (B) Distributions of brain-age gaps, grouped by pubertal stage (parent-report). Means for each group are represented with vertical lines. (C) Distributions of brain-age gaps, grouped by quartile of cognition scores, compared to others in the sample. Only the highest and lowest quartiles are shown. Means for each group are represented with vertical lines.

In the baseline wave (9-11 years old), a higher/more positive BrainAGE was related to more advanced youth- and parent-report pubertal development (Table 4). For youth-report pubertal development, (*b_youth_* = 0.25, *se* = 0.05, *p* < .001) (Fig. 2A). For parent-report pubertal development, (*b_parent_* = 0.24, *se* = 0.04, *p* < .001) (Fig. 2B). Additionally, a higher/more positive BrainAGE was also related to lower cognition scores (*b_cog_* = -0.003, *se* = 0.002, *p* < .001 in both samples) (Fig. 2C).

**Fig. 3.**
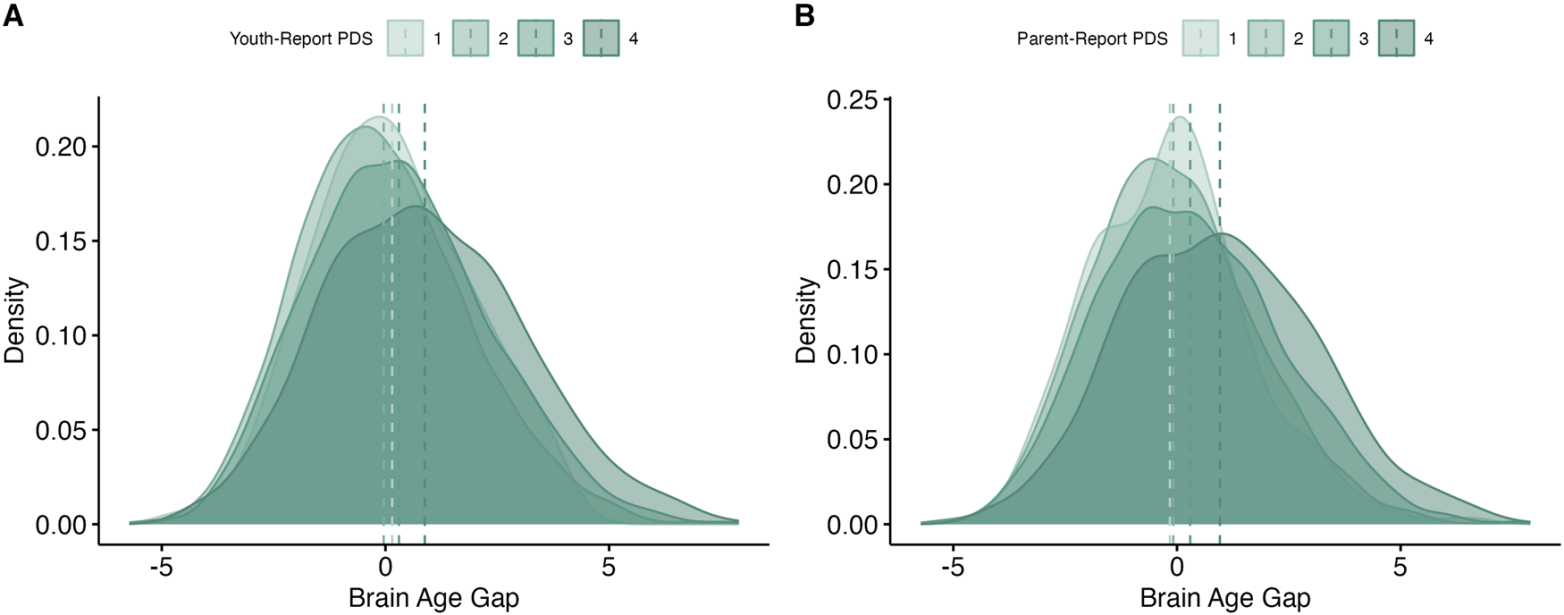
Results: Previously validated model, follow-up sample. (A) Distributions of brain-age gaps, grouped by pubertal stage (youth-report). Means for each group are represented with vertical lines. (B) Distributions of brain-age gaps, grouped by pubertal stage (parent-report). Means for each group are represented with vertical lines.

In the 2-year follow-up, a higher/more positive BrainAGE was related to more advanced youth- and parent-report pubertal development. For youth-report pubertal development, (*b_youth_* = 0.50, *se* = 0.04, *p *< .001) (Fig. 3A). For parent-report pubertal development, (*b_parent_* = 0.58, *se* = 0.03, *p* < .001 in both samples) (Fig. 3B). As discussed above, composite cognition scores were not available at the 2-year follow-up.

### 2.2 Study 2

#### 2.2.1 Participants

##### 2.2.1.1 Samples and models

###### 2.2.1.1.1 Model training and analyses

Adolescents included in the model training, testing, and analyses were participants in the Adolescent Brain Cognitive Development (ABCD) Study, described in Study 1. Data included in Study 2: Baseline were from the baseline sample (9-11 years old) and are identical to the baseline sample described in Study 1. Data included in Study 2: Follow-Up were from the 2-year follow-up (10-13 years old) and are identical to the follow-up sample described in Study 1.

###### 2.2.1.1.2 MRI acquisition

Detailed imaging procedures can be found in Casey et al. (2018) and Hagler et al. (2019). Harmonized protocols were used across 21 sites to collect imaging data using one of three 3 T scanner platforms (Phllips, Siemens, or GE) and a 32-channel head coil. Three-dimensional T1-weighted scans were collected with 1-mm voxel resolution.

#### 2.2.2 Materials & methods

##### 2.2.2.1 MRI processing and model features

The models were trained on and used to predict BrainAGE from the same 175 structural features used in the prediction for Study 1. A complete list of the features used in the training and prediction of Study 2 is available in Supplementary Table 1. As in Study 1, the longCombat package was used to harmonize multisite data.

##### 2.2.2.2 BrainAGE prediction

For both Study 2 models, available data were split into training, validation, and test sets. For the baseline model, half of the sample (5,701 participants) were reserved for the test set. Of the remaining 5,701 participants, 80% (*N* = 4,559) were used for model training, and 20% (*N* = 1,142) were reserved for model validation. For Study 2: Follow-Up, approximately half the sample (3,693 participants) were reserved for the test set. The test set was structured to maximize the number of participants included from the Study 2 baseline test set, enabling further longitudinal analyses. Of the remaining 3,739 participants, 80% (*N* = 2989) were used for model training, and 20% (*N* = 750) were reserved for model validation.

Model training followed the same procedures as the Study 1 model, originally described in Drobinin et al. (2022). Ten-fold cross-validation was repeated 10 times, stratified by scan age. Model training was performed using tidymodels (version 0.2.0; Kuhn & Wickham, 2020), with the XGBoost ML algorithm (version 1.5.2.1; Chen & Guestrin, 2016). Scan age was predicted using the cortical and subcortical features in Supplementary Table 1. The best model was the one with the smallest MAE (mean absolute error), which was expressed in years. Following model validation, the brain-age gap was calculated in the hold-out sample by subtracting the actual scan age/chronological age from the predicted age. Descriptive statistics for BrainAGE predictions and maturational metrics are available in Table 3.

#### 2.2.3 Results

##### 2.2.3.1 BrainAGE model performance

###### 2.2.3.1.1 Model 2: baseline

**Fig. 4.**
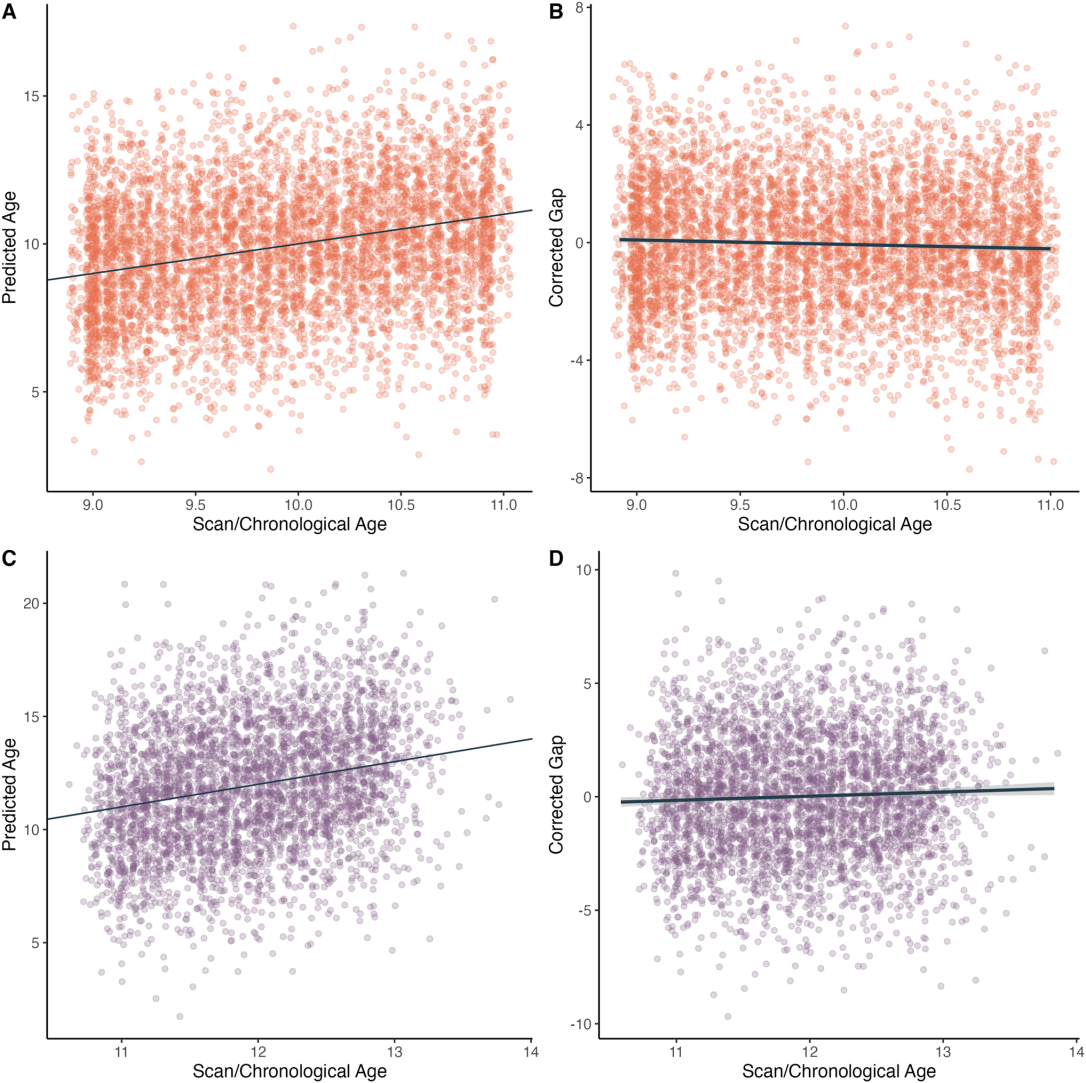
Age versus model predictions for the novel models. (A) Scan/chronological age versus model-predicted age for the baseline sample. The line represents an exact relationship between chronological age and predicted age. (B) Scan/chronological age versus the age-corrected brain-age gap estimate (BrainAGE), for the baseline sample. The regression line shows a slight age-related bias, with younger participants predicted to be slightly older than their chronological age. (C) Similar to (A), scan/chronological age versus predicted age for the follow-up sample. (D) Similar to (B), scan/chronological age versus the age-corrected brain-age gap for the follow-up sample. Again, the regression line shows a slight amount of age bias.

The best baseline model performed with an MAE of 0.14 in cross-validation. In our hold-out validation sample, the model performed with an MAE of 0.49. Finally, in the sample reserved for analyses, the model performed with an MAE of 0.49 (uncorrected for age bias) and 0.86 (corrected). Model performance is shown in Figure 4A,B. Model performance was improved over existing models, including the model used in Study 1. The model showed a slight age bias, again due to the effect of regression on the mean. Corrected BrainAGE had a correlation of -0.20 with chronological age (Fig. 4B).

###### 2.2.3.1.2 Model 2: follow-up

The best baseline model performed with an MAE of 0.53 in cross-validation. In our hold-out validation sample, the model performed with an MAE of 0.53. Finally, in the sample reserved for analyses, the model performed with an MAE of 0.52 (uncorrected for age bias) and 2.2 (corrected). Model performance is shown in Figure 4C,D. As with the baseline model, the novel follow-up model performed with similar accuracy to existing adolescent models. The model showed a slight age bias, again due to the effect of regression on the mean. Corrected BrainAGE had a correlation of 0.04 with chronological age (Fig. 4D).

##### 2.2.3.2 Neuroanatomical contributions

Top contributors to the models were determined using the vip R package (version 0.4.1), and visualized with the ggseg R package (version 1.6.5; Mowinckel & Vidal-Piñeiro, 2020). For models built using xgboost, the vip package determines variable importance by ranking the absolute magnitude of the estimated coefficients.

###### 2.2.3.2.1 Baseline model

The top contributors to the baseline model included the volume of the brainstem, mid-posterior corpus callosum, left posterior cingulate, right cuneus, left postcentral gyrus, and right isthmus cingulate.

**Fig. 5.**
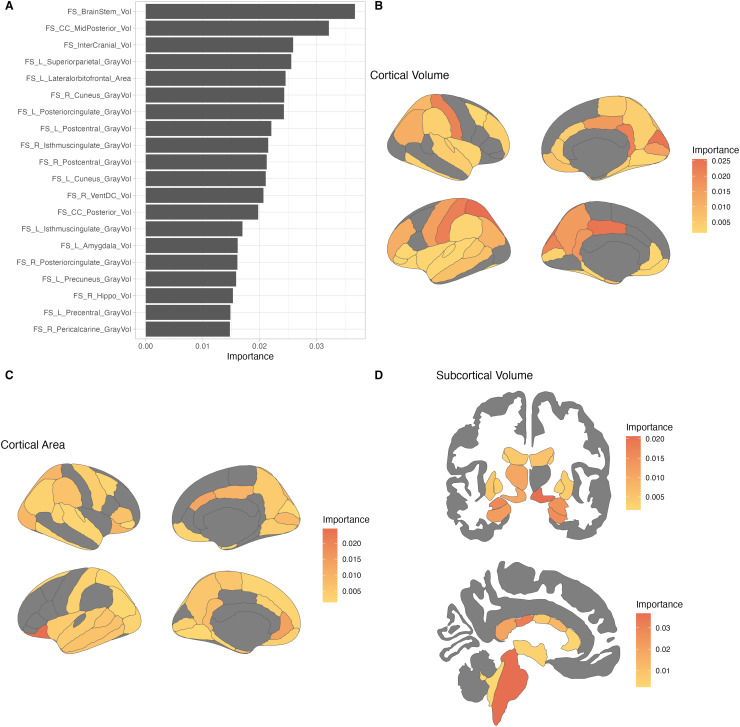
Neuroanatomical contributors to the novel model (baseline). (A) Top 20 features ranked by importance to the model, across domains. (B) Variable importance of cortical volume features, visualized on 2-dimensional brain. (C) Variable importance of cortical area features, visualized on 2-dimensional brain. (D) Variable importance of subcortical features, visualized on 2-dimensional brain. Top: Coronal view. Bottom: Sagittal view.

Figure 5 shows the cortical and subcortical features with the greatest importance to the model prediction.

To contextualize our model features, we compared our findings to those of past adolescent BrainAGE models, including those of Drobinin et al. (2022) and Kelly et al. (2022). While BrainAGE model features cannot always be directly compared due to different brain parcellations and reporting methods, there were a number of high-contributing features in the novel model that are shared with those found in existing work. Shared with Drobinin et al. (2022), we found that the brainstem, superior parietal, isthmus cingulate, ventral diencephalon, amygdala, hippocampus, and posterior corpus callosum were high contributors to the model.

Additionally, we compared our results with those of the model from Kelly et al. (2022) and found that both models shared volumes of frontal, temporal, and parietal regions, the brainstem as high contributors.

###### 2.2.3.2.2 Follow-up

As in the baseline model, top contributors to the model were determined using the vip R package (version 0.4.1). The top contributors to the model included the intercranial volume, as well as the volume of the brainstem, mid-posterior and posterior corpus callosum, right isthmus cingulate, right inferior parietal, and left rostral middle frontal regions. Generally, volume measurements contributed more highly to the model than surface area.

Many of these regions were also top contributors to the baseline model, including the brainstem, intercranial volume, and corpus callosum. New high contributors included the right inferior parietal, rostral middle frontal, and lateral ventricle volumes, as well as the caudal anterior cingulate area. Compared to the Drobinin et al. (2022) model, shared regions included the brainstem, corpus callosum, amygdala, inferior parietal, and isthmus cingulate. Shared regions with the Kelly et al. (2022) included the brainstem, ventricles, as well as frontal, temporal, and parietal volumes.

##### 2.2.3.3 BrainAGE and maturational metrics

###### 2.2.3.3.1 Baseline

**Fig. 6.**
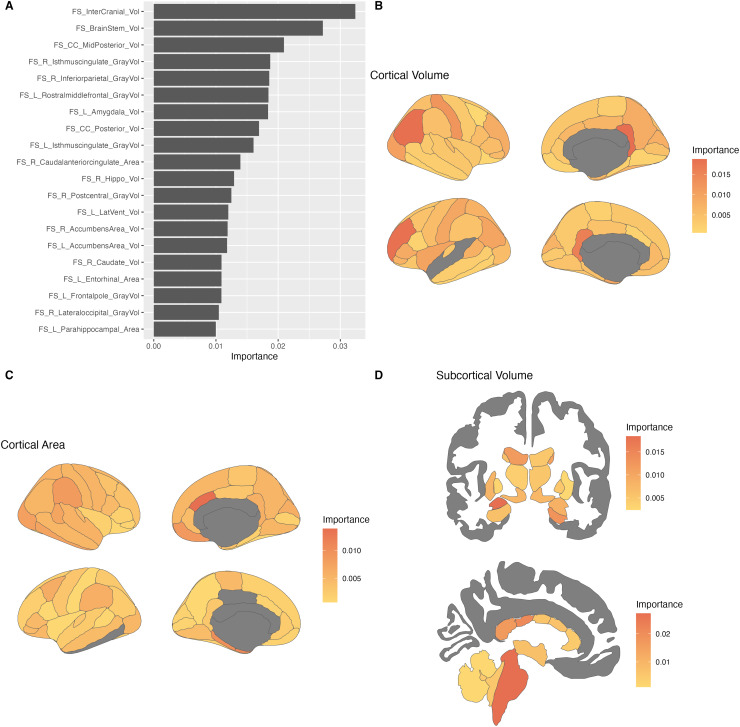
Neuroanatomical contributors to the novel model (follow-up). (A) Top 20 features ranked by importance to the model, across domains. (B) Variable importance of cortical volume features, visualized on 2-dimensional brain. (C) Variable importance of cortical area features, visualized on 2-dimensional brain. (D) Variable importance of subcortical features, visualized on 2-dimensional brain. Top: Coronal view. Bottom: Sagittal view.

**Fig. 7.**
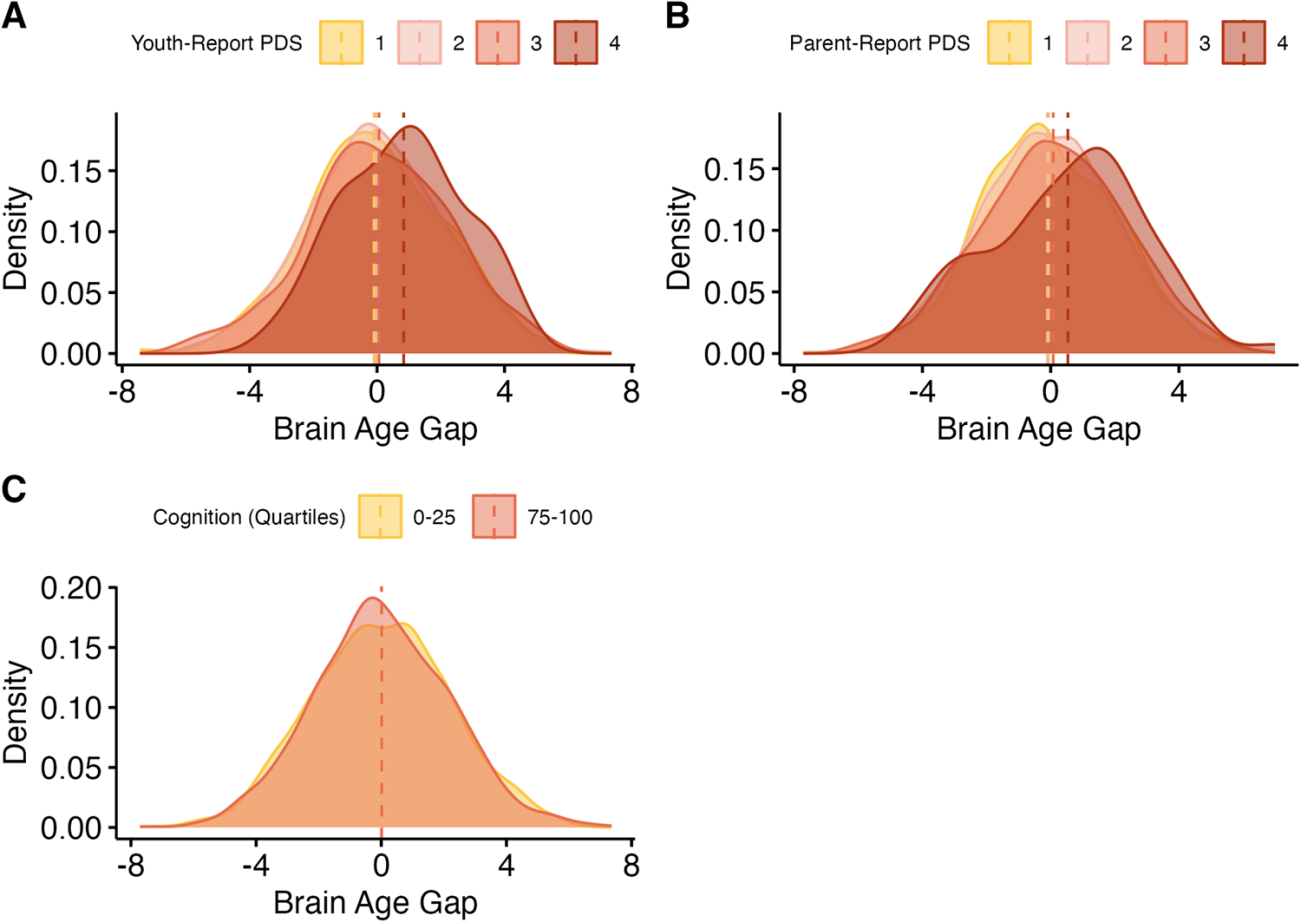
Results: Novel model (baseline). (A) Distributions of brain-age gaps, grouped by pubertal stage (youth-report). Means for each group are represented with vertical lines. (B) Distributions of brain-age gaps, grouped by pubertal stage (parent-report). Means for each group are represented with vertical lines. (C) Distributions of brain-age gaps, grouped by quartile of cognition scores, compared to others in the sample. Only the highest and lowest quartiles are shown. Means for each group are represented with vertical lines.

A higher BrainAGE was related to more advanced youth- and parent-report pubertal development (Table 4). For youth-report pubertal development, (*b_youth_* = 0.31, *se* = 0.09, *p* < .001) (Fig. 7A). For parent-report pubertal development, (*b_parent_* = 0.19, *se* = 0.06, *p* < .001) (Fig. 7B). In addition, a higher/more positive BrainAGE was related to higher cognition scores (*b_cog_* = 0.002, *se* = 0.003, *p* = .005) (Fig. 6C).

###### 2.2.3.3.2 Follow-up

**Fig. 8.**
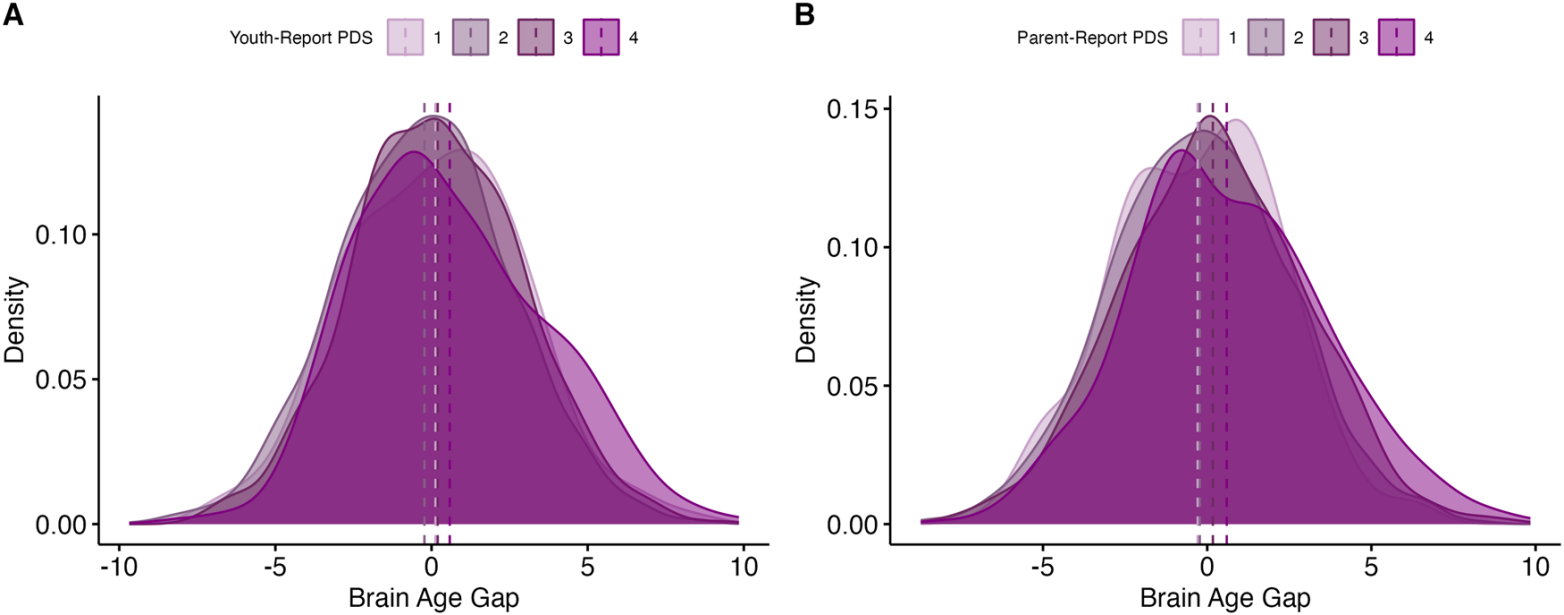
Results: Novel model (follow-up). (A) Distributions of brain-age gaps, grouped by pubertal stage (youth-report). Means for each group are represented with vertical lines. (B) Distributions of brain-age gaps, grouped by pubertal stage (parent-report). Means for each group are represented with vertical lines.

A higher BrainAGE was related to more advanced youth- and parent-report pubertal development (Table 4). For youth-report pubertal development, (*b_youth_* = 0.39, *se* = 0.08, *p *< .001) (Fig. 8A). For parent-report pubertal development, (*b_parent_* = 0.42, *se* = 0.07, *p *< .001) (Fig. 8B).

##### 2.2.3.4 Longitudinal analyses

###### 2.2.3.4.1 Methods

To examine the stability of BrainAGE measurements over time, we calculated the intraclass correlation (ICC) between BrainAGE at baseline and 2-year follow-up for both studies. The ICC quantifies the variance between people and the variance over time, where an ICC of 0 would indicate that all variance is within-person, and an ICC of 1 would indicate that all variance in between-persons (Little et al., 2015). Only participants with BrainAGE estimates at both timepoints were included in these analyses. Using the Study 1 gap predictions, 7,431 participants had data at both timepoints. Using the Study 2 gap predictions, 3,692 participants had estimates at both timepoints.

###### 2.2.3.4.2 Results

**Fig. 9.**
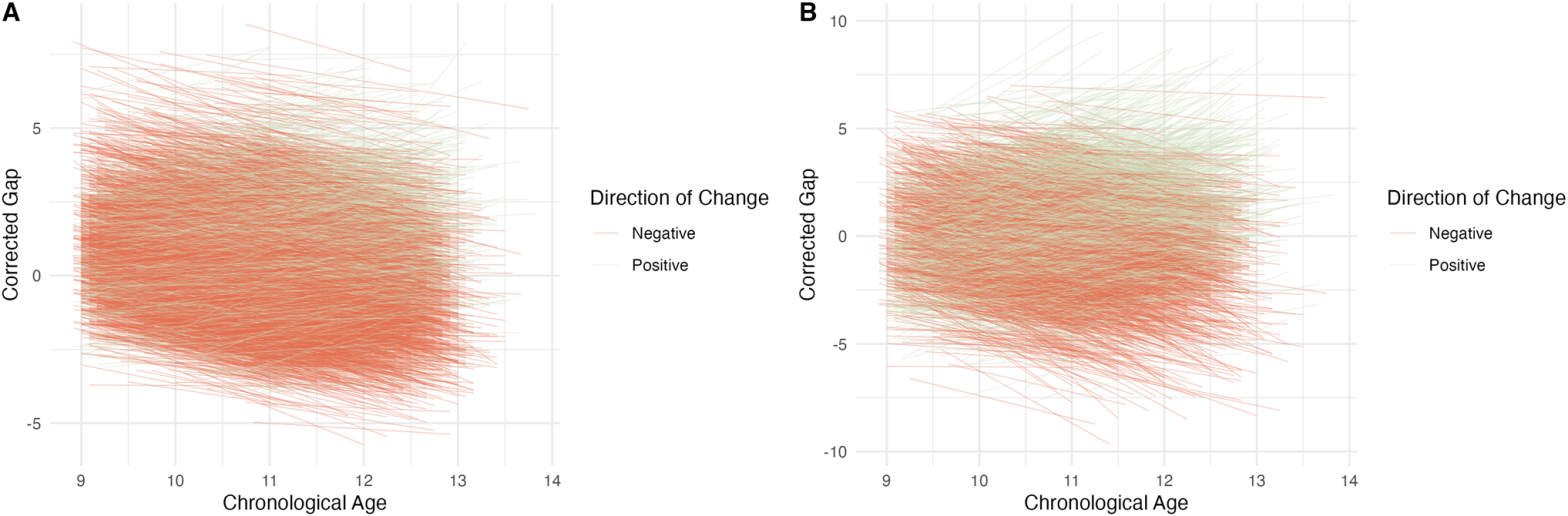
Results: Longitudinal changes in gap estimates. (A) Individual trajectories of brain-age gaps, where both gaps were predicted using the same model (Study 1). Trajectories are color-coded by whether the estimated gap increased or decreased between timepoints. (B) Individual trajectories of brain-age gaps, where the gap at each timepoint was predicted from a timepoint-specific model (Study 2). Trajectories are color-coded by whether the estimated gap increased or decreased between timepoints.

BrainAGE showed moderate to large ICCs across two timepoints in early adolescence. For Study 1, the ICC between BrainAGE estimates was 0.7 (Fig. 9A). For Study 2, the ICC between BrainAGE estimates was 0.53 (Fig. 9B).

## 3 Discussion

The current study used three BrainAGE models—one previously validated on a wide age range across adolescence and two trained on specific, narrow age ranges in early adolescence—to quantify the relationship between BrainAGE and metrics of pubertal and cognitive development. Using the previously validated model, we established that BrainAGE is positively related to both youth- and parent-report pubertal development, and this relationship holds across two age ranges (9-11, 10-13 years). This association was replicated using our novel models created from independent samples of 9-11 and 10-13 year olds.

The current paper builds on our existing understanding of the relationship between puberty/maturational metrics and BrainAGE in adolescence. Notably, the relationship between pubertal development and BrainAGE was replicated using a model trained on the specific age range of our analysis sample, which featured a lower MAE and reduced age bias. Furthermore, these models and their accompanying training code are available to other researchers to enable further investigation of BrainAGE in early adolescence.

While the relationship between BrainAGE and NIH Toolbox Cognition scores was consistently negatively related across subsamples of estimates derived from the validated model, these results were in conflict with the positive relationship between BrainAGE and cognition scores found in the estimates derived from the novel model. NIH Toolbox Cognition summary scores were not calculated for the 2-year follow-up data within ABCD Release 4.0, so it was not possible to examine whether these relationships replicate across waves.

Additionally, we examined longitudinal change in BrainAGE using age-specific models, and found moderate to large ICCs across two timepoints in early adolescence. Stability in BrainAGE was higher when using the same model to predict age at multiple timepoints, compared to using separate models for each timepoint. This indicates that timepoint-specific models may capture more changes that occur in specific developmental periods, but may also be more difficult to interpret between models. Additionally, there was individual variation in the direction of change in BrainAGE, and there were differences in the degree of variation of trajectories between models.

The results of the current study provide initial evidence that BrainAGE tracks with some metrics of maturation, including pubertal development. However, the conflicting results between BrainAGE and cognition lead us to question the utility of these models for non-biological processes. The improvement in MAE between the age groups may be due to the fact that the younger age group was on the lower end/extreme of the age range used to train the model, which might have had fewer participants in the training set than in the older group, so the model would be more accurate for the older age group. Potential sources of the improved MAE (mean absolute error) could be the large amount of data, the narrow age range used in both training and testing (a novel feature of the study), and the use of independent samples of data derived from the same study (ABCD) for training, testing, and prediction in Study 2. In line with our findings, Ball et al. (2017) suggested that image-based models predict most accurately during periods when the rate of anatomical change is greatest. The current study provides a novel BrainAGE model trained on a large sample of early adolescents, which can be used with developmental samples such as ABCD. The novel model is available for use and can be found at https://github.com/LucyWhitmore/BrainAGE-Maturation.

The current study used composite cognition scores which collapse across multiple domains. To accurately assess the relationship between BrainAGE and cognition, we may need to look at individual domains that show dramatic change in early adolescence, such as executive function (Tervo-Clemmens et al., 2022, preprint). While the NIH Toolbox Cognition Battery is generally sensitive to change over our age range of interest, specific domains may be more or less strongly related to BrainAGE (Bauer & Zelazo, 2013).

While the NIH Toolbox is intended to be used over a lifespan age range, measures targeted specifically at constructs that change in early adolescence may provide a clearer view of how BrainAGE relates to cognition in this age range. Furthermore, the lack of complete composite cognition scores at the 2-year follow-up limits our ability to examine the relationship between BrainAGE and cognition in multiple age bands. In future work, we may be able to see whether the inconsistent relationship persists in older adolescents.

Additionally, a robust relationship was observed between BrainAGE and puberty across subsamples, waves, models, and reporting methods. These results provide some evidence that BrainAGE tracks with known metrics of pubertal maturation. The relationship between BrainAGE and pubertal development strengthened between waves, possibly due to increased representation of later pubertal stages in the older age range. In future work, these analyses can be extended to track the relationship between BrainAGE and puberty within different age bands in adolescence and illustrate when BrainAGE and puberty are most closely tied. Additionally, youth- and parent-report pubertal development are known to vary in their reliability across age, with parent-report being more reliable at younger ages, and youth-report being more reliable at older ages (Terry et al., 2016). In the current study, we have shown that both reporting methods are consistently linked to BrainAGE. Future work may be able to illustrate when, or if, one method of reporting is no longer associated with BrainAGE.

To make matters more complicated, there are reciprocal connections between many of the constructs that have been related to BrainAGE. Here, we showed that BrainAGE is related to pubertal development, and past work has shown BrainAGE is related to psychopathology as well as adversity and, in adults, early life events such as low birth weight (Drobinin et al., 2022; Vidal-Pineiro et al., 2021). However, earlier pubertal timing has also been related to increased risk for psychopathology, and both psychopathology and early pubertal timing are related to environmental factors (Barendse et al., 2022; Graber, 2013; Graber et al., 1995; Mclaughlin et al., 2012). Therefore, it remains difficult to untangle these relationships and mechanisms.

Additionally, more longitudinal BrainAGE work is needed to fully characterize the relationship between BrainAGE and maturational metrics. The current study provides evidence that BrainAGE is positively correlated with pubertal development in early adolescence, but future work should investigate the potentially changing relationship between puberty and BrainAGE in a longitudinal framework, and over a wider range of adolescence.

Our longitudinal analyses showed moderate to large ICCs between BrainAGE at two timepoints, and a larger ICC for BrainAGE predicted by the same model at different waves. However, future work should examine how changes in BrainAGE over time, including changes in direction, relate to outcomes. Furthermore, there is a need for more work considering the impact of age correction and age bias on adolescent BrainAGE models. As discussed earlier, these models are subject to regression to the mean, where younger participants are predicted to be older, and older participants predicted to be younger. While most BrainAGE models are bias corrected, this correction is not perfect. This presents potential issues for longitudinal analyses, where trajectories of BrainAGE may be more likely to be negative when using the same model across waves as younger participants in the model have a larger, positive BrainAGE.

Additionally, many of the features used to train BrainAGE models have huge variability and individual differences in adolescence, and brain maturity is more related to trajectories of brain development than overall values, which is not accounted for in most BrainAGE models (Mills et al., 2021). In current models, a participant may be considered younger or older because of the starting size of their brain, while they are in fact still following a normative developmental trajectory.

In summary, BrainAGE is a powerful tool that has promise as a clinical biomarker and as a tool to inform basic research. However, as BrainAGE gains traction within the field of developmental cognitive neuroscience, we must ensure that we are careful about our interpretations of its predictions and associations and that we are confident in what we are actually measuring when we talk about brain maturity. As the results of brain maturity studies have implications for legal and other policy domains, it is vital that researchers provide contextualization for BrainAGE and be clear about what we can and cannot infer about different forms of maturity. BrainAGE is a powerful and promising tool, but we need to ensure that we have a strong theoretical background before widely incorporating it into studies of adolescent development and maturation.

## 4 Conclusions

The present study demonstrates that BrainAGE is positively correlated with pubertal development in a sample of early adolescents, and that this result is replicable across subsamples, models, and multiple age bands. However, the relationship between BrainAGE and cognition in early adolescence remains unclear. Future work should examine these relationships in later adolescence and utilize cognition measures that are optimized to examine changes across adolescence.

## Supplementary Material

Supplementary Material

## Data Availability

Analysis scripts can be found at: https://github.com/LucyWhitmore/BrainAGE-Maturation The publicly available existing model, as well as training and testing scripts, can be found at: https://github.com/GitDro/DevelopmentalBrainAge. Data are part of the ABCD 4.0 Data Release, and are available with an NIH Data Use Certification. The 4.0 release can be found at: http://dx.doi.org/10.15154/1523041

## References

[b1] Anokhin, A. P., Luciana, M., Banich, M., Barch, D., Bjork, J. M., Gonzalez, M. R., Gonzalez, R., Haist, F., Jacobus, J., Lisdahl, K., McGlade, E., McCandliss, B., Nagel, B., Nixon, S. J., Tapert, S., Kennedy, J. T., & Thompson, W. (2022). Age-related changes and longitudinal stability of individual differences in ABCD neurocognition measures. Developmental Cognitive Neuroscience, 54, 101078. 10.1016/j.dcn.2022.101078 35123342 PMC9019835

[b3] Ball, G., Adamson, C., Beare, R., & Seal, M. L. (2017). Modelling neuroanatomical variation during childhood and adolescence with neighbourhood-preserving embedding. Scientific Reports, 7(1), Article 1. 10.1038/s41598-017-18253-6 PMC5736651 29259302

[b4] Ball, G., Kelly, C. E., Beare, R., & Seal, M. L. (2021). Individual variation underlying brain age estimates in typical development. NeuroImage, 235, 118036. 10.1016/j.neuroimage.2021.118036 33838267

[b5] Barch, D. M., Albaugh, M. D., Avenevoli, S., Chang, L., Clark, D. B., Glantz, M. D., Hudziak, J. J., Jernigan, T. L., Tapert, S. F., Yurgelun-Todd, D., Alia-Klein, N., Potter, A. S., Paulus, M. P., Prouty, D., Zucker, R. A., & Sher, K. J. (2018). Demographic, physical and mental health assessments in the adolescent brain and cognitive development study: Rationale and description. Developmental Cognitive Neuroscience, 32, 55–66. 10.1016/j.dcn.2017.10.010 29113758 PMC5934320

[b6] Barendse, M. E. A., Byrne, M. L., Flournoy, J. C., McNeilly, E. A., Guazzelli Williamson, V., Barrett, A.-M. Y., Chavez, S. J., Shirtcliff, E. A., Allen, N. B., & Pfeifer, J. H. (2022). Multimethod assessment of pubertal timing and associations with internalizing psychopathology in early adolescent girls. Journal of Psychopathology and Clinical Science, 131(1), 14–25. 10.1037/abn0000721 34941314 PMC9439585

[b7] Bauer, P. J., & Zelazo, P. D. (2013). Ix. Nih Toolbox cognition battery (cb): Summary, conclusions, and implications for cognitive development. Monographs of the Society for Research in Child Development, 78(4), 133–146. 10.1111/mono.12039 23952207

[b8] Beer, J. C., Tustison, N. J., Cook, P. A., Davatzikos, C., Sheline, Y. I., Shinohara, R. T., & Linn, K. A. (2020). Longitudinal ComBat: A method for harmonizing longitudinal multi-scanner imaging data. NeuroImage, 220, 117129. 10.1016/j.neuroimage.2020.117129 32640273 PMC7605103

[b9] Bethlehem, R. a. I., Seidlitz, J., White, S. R., Vogel, J. W., Anderson, K. M., Adamson, C., Adler, S., Alexopoulos, G. S., Anagnostou, E., Areces-Gonzalez, A., Astle, D. E., Auyeung, B., Ayub, M., Bae, J., Ball, G., Baron-Cohen, S., Beare, R., Bedford, S. A., Benegal, V.,… Alexander-Bloch, A. F. (2022). Brain charts for the human lifespan. Nature, 604(7906), Article 7906. 10.1038/s41586-022-04554-y PMC9021021 35388223

[b10] Biondo, F., Jewell, A., Pritchard, M., Aarsland, D., Steves, C. J., Mueller, C., & Cole, J. H. (2022). Brain-age is associated with progression to dementia in memory clinic patients. NeuroImage: Clinical, 36, 103175. 10.1016/j.nicl.2022.103175 36087560 PMC9467894

[b12] Bleck, T. P., Nowinski, C. J., Gershon, R., & Koroshetz, W. J. (2013). What is the NIH Toolbox, and what will it mean to neurology? Neurology, 80(10), 874–875. 10.1212/WNL.0b013e3182872ea0 23460616

[b13] Brouwer, R. M., Schutte, J., Janssen, R., Boomsma, D. I., Hulshoff Pol, H. E., & Schnack, H. G. (2021). The speed of development of adolescent brain age depends on sex and is genetically determined. Cerebral Cortex, 31(2), 1296–1306. 10.1093/cercor/bhaa296 33073292 PMC8204942

[b14] Brown, T. T., Kuperman, J. M., Chung, Y., Erhart, M., McCabe, C., Hagler, D. J., Venkatraman, V. K., Akshoomoff, N., Amaral, D. G., Bloss, C. S., Casey, B. J., Chang, L., Ernst, T. M., Frazier, J. A., Gruen, J. R., Kaufmann, W. E., Kenet, T., Kennedy, D. N., Murray, S. S.,… Dale, A. M. (2012). Neuroanatomical assessment of biological maturity. Current Biology, 22(18), 1693–1698. 10.1016/j.cub.2012.07.002 22902750 PMC3461087

[b16] Callaghan, B. L., & Tottenham, N. (2016). The stress acceleration hypothesis: Effects of early-life adversity on emotion circuits and behavior. Current Opinion in Behavioral Sciences, 7, 76–81. 10.1016/j.cobeha.2015.11.018 29644262 PMC5890821

[b17] Casey, B. J., Cannonier, T., Conley, M. I., Cohen, A. O., Barch, D. M., Heitzeg, M. M., Soules, M. E., Teslovich, T., Dellarco, D. V., Garavan, H., Orr, C. A., Wager, T. D., Banich, M. T., Speer, N. K., Sutherland, M. T., Riedel, M. C., Dick, A. S., Bjork, J. M., Thomas, K. M.,… Dale, A. M. (2018). The Adolescent Brain Cognitive Development (ABCD) study: Imaging acquisition across 21 sites. Developmental Cognitive Neuroscience, 32, 43–54. 10.1016/j.dcn.2018.03.001 29567376 PMC5999559

[b18] Chen, T., & Guestrin, C. (2016). XGBoost: A scalable tree boosting system. Proceedings of the 22nd ACM SIGKDD International Conference on Knowledge Discovery and Data Mining, 785–794. 10.1145/2939672.2939785

[b20] Chung, Y., Addington, J., Bearden, C. E., Cadenhead, K., Cornblatt, B., Mathalon, D. H., McGlashan, T., Perkins, D., Seidman, L. J., Tsuang, M., Walker, E., Woods, S. W., McEwen, S., van Erp, T. G. M., Cannon, T. D., & for the North American Prodrome Longitudinal Study (NAPLS) Consortium and the Pediatric Imaging, N., and Genetics (PING) Study Consortium. (2018). Use of machine learning to determine deviance in neuroanatomical maturity associated with future psychosis in youths at clinically high risk. JAMA Psychiatry, 75(9), 960–968. 10.1001/jamapsychiatry.2018.1543 29971330 PMC6142910

[b21] Cromer, J. A., Schembri, A. J., Harel, B. T., & Maruff, P. (2015). The nature and rate of cognitive maturation from late childhood to adulthood. Frontiers in Psychology, 6. https://www.frontiersin.org/articles/10.3389/fpsyg.2015.00704 10.3389/fpsyg.2015.00704 PMC4445246 26074853

[b22] Cropley, V. L., Tian, Y., Fernando, K., Mansour L., S., Pantelis, C., Cocchi, L., & Zalesky, A. (2021). Brain-predicted age associates with psychopathology dimensions in youths. Biological Psychiatry: Cognitive Neuroscience and Neuroimaging, 6(4), 410–419. 10.1016/j.bpsc.2020.07.014 32981878

[b23] Davidson, M. C., Amso, D., Anderson, L. C., & Diamond, A. (2006). Development of cognitive control and executive functions from 4 to 13 years: Evidence from manipulations of memory, inhibition, and task switching. Neuropsychologia, 44(11), 2037–2078. 10.1016/j.neuropsychologia.2006.02.006 16580701 PMC1513793

[b24] Dehestani, N., Whittle, S., Vijayakumar, N., & Silk, T. J. (2023). Developmental brain changes during puberty and associations with mental health problems. Developmental Cognitive Neuroscience, 60, 101227. 10.1016/j.dcn.2023.101227 36933272 PMC10036507

[b28] Drobinin, V., Gestel, H. V., Helmick, C. A., Schmidt, M. H., Bowen, C. V., & Uher, R. (2022). The developmental brain age is associated with adversity, depression, and functional outcomes among adolescents. Biological Psychiatry: Cognitive Neuroscience and Neuroimaging, 7(4), 406–414. 10.1016/j.bpsc.2021.09.004 34555562

[b29] Erus, G., Battapady, H., Satterthwaite, T. D., Hakonarson, H., Gur, R. E., Davatzikos, C., & Gur, R. C. (2015). Imaging patterns of brain development and their relationship to cognition. Cerebral Cortex, 25(6), 1676–1684. 10.1093/cercor/bht425 24421175 PMC4428302

[b30] Franke, K., & Gaser, C. (2012). Longitudinal changes in individual BrainAGE in healthy aging, mild cognitive impairment, and Alzheimer’s disease. GeroPsych: The Journal of Gerontopsychology and Geriatric Psychiatry, 25, 235–245. 10.1024/1662-9647/a000074

[b31] Franke, K., & Gaser, C. (2019). Ten years of BrainAGE as a neuroimaging biomarker of brain aging: What insights have we gained? Frontiers in Neurology, 10. https://www.frontiersin.org/articles/10.3389/fneur.2019.00789 10.3389/fneur.2019.00789 PMC6702897 31474922

[b32] Franke, K., Hagemann, G., Schleussner, E., & Gaser, C. (2015). Changes of individual BrainAGE during the course of the menstrual cycle. NeuroImage, 115, 1–6. 10.1016/j.neuroimage.2015.04.036 25913700

[b33] Franke, K., Luders, E., May, A., Wilke, M., & Gaser, C. (2012). Brain maturation: Predicting individual BrainAGE in children and adolescents using structural MRI. NeuroImage, 63(3), 1305–1312. 10.1016/j.neuroimage.2012.08.001 22902922

[b34] Franke, K., Ziegler, G., Klöppel, S., & Gaser, C. (2010). Estimating the age of healthy subjects from T1-weighted MRI scans using kernel methods: Exploring the influence of various parameters. NeuroImage, 50(3), 883–892. 10.1016/j.neuroimage.2010.01.005 20070949

[b36] Garavan, H., Bartsch, H., Conway, K., Decastro, A., Goldstein, R. Z., Heeringa, S., Jernigan, T., Potter, A., Thompson, W., & Zahs, D. (2018). Recruiting the ABCD sample: Design considerations and procedures. Developmental Cognitive Neuroscience, 32, 16–22. 10.1016/j.dcn.2018.04.004 29703560 PMC6314286

[b37] Gershon, R. C., Wagster, M. V., Hendrie, H. C., Fox, N. A., Cook, K. F., & Nowinski, C. J. (2013). NIH Toolbox for assessment of neurological and behavioral function. Neurology, 80(11 Supplement 3), S2–S6. 10.1212/WNL.0b013e3182872e5f 23479538 PMC3662335

[b38] Goddings, A.-L., Mills, K. L., Clasen, L. S., Giedd, J. N., Viner, R. M., & Blakemore, S.-J. (2014). The influence of puberty on subcortical brain development. NeuroImage, 88, 242–251. 10.1016/j.neuroimage.2013.09.073 24121203 PMC3991320

[b39] Graber, J. A. (2013). Pubertal timing and the development of psychopathology in adolescence and beyond. Hormones and Behavior, 64(2), 262–269. 10.1016/j.yhbeh.2013.04.003 23998670

[b40] Graber, J. A., Brooks-Gunn, J., & Warren, M. P. (1995). The antecedents of menarcheal age: Heredity, family environment, and stressful life events. Child Development, 66(2), 346–359. 10.2307/1131582 7750370

[b41] Gur, R. C., Richard, J., Calkins, M. E., Chiavacci, R., Hansen, J. A., Bilker, W. B., Loughead, J., Connolly, J. J., Qiu, H., Mentch, F. D., Abou-Sleiman, P. M., Hakonarson, H., & Gur, R. E. (2012). Age group and sex differences in performance on a computerized neurocognitive battery in children age 8–21. Neuropsychology, 26(2), 251–265. 10.1037/a0026712 22251308 PMC3295891

[b42] Hagler, D. J., Hatton, S. N., Cornejo, M. D., Makowski, C., Fair, D. A., Dick, A. S., Sutherland, M. T., Casey, B., Barch, D. M., Harms, M. P., Watts, R., Bjork, J. M., Garavan, H. P., Hilmer, L., Pung, C. J., Sicat, C. S., Kuperman, J., Bartsch, H., Xue, F.,… Dale, A. M. (2019). Image processing and analysis methods for the Adolescent Brain Cognitive Development Study. NeuroImage, 202, 116091. 10.1016/j.neuroimage.2019.116091 31415884 PMC6981278

[b43] Han, L. K. M., Dinga, R., Hahn, T., Ching, C. R. K., Eyler, L. T., Aftanas, L., Aghajani, M., Aleman, A., Baune, B. T., Berger, K., Brak, I., Filho, G. B., Carballedo, A., Connolly, C. G., Couvy-Duchesne, B., Cullen, K. R., Dannlowski, U., Davey, C. G., Dima, D.,… Schmaal, L. (2021). Brain aging in major depressive disorder: Results from the ENIGMA major depressive disorder working group. Molecular Psychiatry, 26(9), Article 9. 10.1038/s41380-020-0754-0 PMC8589647 32424236

[b44] Han, L. K. M., Dinga, R., Leenings, R., Hahn, T., Cole, J. H., Aftanas, L. I., Amod, A. R., Besteher, B., Colle, R., Corruble, E., Couvy-Duchesne, B., Danilenko, K. V., Fuentes-Claramonte, P., Gonul, A. S., Gotlib, I. H., Goya-Maldonado, R., Groenewold, N. A., Hamilton, P., Ichikawa, N.,… Schmaal, L. (2022). A large-scale ENIGMA multisite replication study of brain age in depression. Neuroimage: Reports, 2(4), 100149. 10.1016/j.ynirp.2022.100149 PMC12172781 40567563

[b46] Herting, M. M., Gautam, P., Spielberg, J. M., Dahl, R. E., & Sowell, E. R. (2015). A longitudinal study: Changes in cortical thickness and surface area during pubertal maturation. PLoS One, 10(3), e0119774. 10.1371/journal.pone.0119774 25793383 PMC4368209

[b47] Herting, M. M., & Sowell, E. R. (2017). Puberty and structural brain development in humans. Frontiers in Neuroendocrinology, 44, 122–137. 10.1016/j.yfrne.2016.12.003 28007528 PMC5612369

[b48] Hodes, R. J., Insel, T. R., Landis, S. C., & Research, O. behalf of the N. B. for N. (2013). The NIH Toolbox: Setting a standard for biomedical research. Neurology, 80(11 Supplement 3), S1–S1. 10.1212/WNL.0b013e3182872e90 PMC3662338 23479536

[b49] Holm, M. C., Leonardsen, E. H., Beck, D., Dahl, A., Kjelkenes, R., de Lange, A.-M. G., & Westlye, L. T. (2023). Linking brain maturation and puberty during early adolescence using longitudinal brain age prediction in the ABCD cohort. Developmental Cognitive Neuroscience, 60, 101220. 10.1016/j.dcn.2023.101220 36841180 PMC9972398

[b50] Kelly, C., Ball, G., Matthews, L. G., Cheong, J. L., Doyle, L. W., Inder, T. E., Thompson, D. K., & Anderson, P. J. (2022). Investigating brain structural maturation in children and adolescents born very preterm using the brain age framework. NeuroImage, 247, 118828. 10.1016/j.neuroimage.2021.118828 34923131

[b51] Khundrakpam, B. S., Tohka, J., & Evans, A. C. (2015). Prediction of brain maturity based on cortical thickness at different spatial resolutions. NeuroImage, 111, 350–359. 10.1016/j.neuroimage.2015.02.046 25731999

[b52] Kuhn, M., & Wickham, H. (2020). Tidymodels: A collection of packages for modeling and machine learning using tidyverse principles. https://www.tidymodels.org

[b53] Le, T. T., Kuplicki, R. T., McKinney, B. A., Yeh, H.-W., Thompson, W. K., Paulus, M. P., Tulsa 1000 Investigators, Aupperle, R. L., Bodurka, J., Cha, Y.-H., Feinstein, J. S., Khalsa, S. S., Savitz, J., Simmons, W. K., & Victor, T. A. (2018). A nonlinear simulation framework supports adjusting for age when analyzing BrainAGE. Frontiers in Aging Neuroscience, 10. https://www.frontiersin.org/articles/10.3389/fnagi.2018.00317 10.3389/fnagi.2018.00317 PMC6208001 30405393

[b54] Lewis, J. D., Evans, A. C., & Tohka, J. (2018). T1 white/gray contrast as a predictor of chronological age, and an index of cognitive performance. NeuroImage, 173, 341–350. 10.1016/j.neuroimage.2018.02.050 29501876

[b55] Little TD , Schnabel KU, Baumert J. (2015). Modeling longitudinal and multilevel data: Practical issues, applied approaches, and specific examples. Psychology Press.

[b56] Luciana, M., Bjork, J. M., Nagel, B. J., Barch, D. M., Gonzalez, R., Nixon, S. J., & Banich, M. T. (2018). Adolescent neurocognitive development and impacts of substance use: Overview of the adolescent brain cognitive development (ABCD) baseline neurocognition battery. Developmental Cognitive Neuroscience, 32, 67–79. 10.1016/j.dcn.2018.02.006 29525452 PMC6039970

[b57] McLaughlin, K. A., Greif Green, J., Gruber, M. J., Sampson, N. A., Zaslavsky, A. M., & Kessler, R. C. (2012). Childhood adversities and first onset of psychiatric disorders in a national sample of US adolescents. Archives of General Psychiatry, 69(11), 1151–1160. 10.1001/archgenpsychiatry.2011.2277 23117636 PMC3490224

[b87] Mills, K. L., Goddings, A.-L., Herting, M. M., Meuwese, R., Blakemore, S.-J., Crone, E. A., Dahl, R. E., Güroglu, B., Raznahan, A., Sowell, E. R., & Tamnes, C. K. (2016). Structural brain development between childhood and adulthood: Convergence across four longitudinal samples. NeuroImage, 141, 273–281. 10.1016/j.neuroimage.2016.07.044 27453157 PMC5035135

[b59] Mills, K. L., Siegmund, K. D., Tamnes, C. K., Ferschmann, L., Wierenga, L. M., Bos, M. G. N., Luna, B., Li, C., & Herting, M. M. (2021). Inter-individual variability in structural brain development from late childhood to young adulthood. NeuroImage, 242, 118450. 10.1016/j.neuroimage.2021.118450 34358656 PMC8489572

[b60] Mowinckel, A. M., & Vidal-Piñeiro, D. (2020). Visualization of brain statistics with R packages ggseg and ggseg3d. Advances in Methods and Practices in Psychological Science, 3(4), 466–483. 10.1177/2515245920928009

[b61] Nguyen, T.-V., McCracken, J., Ducharme, S., Botteron, K. N., Mahabir, M., Johnson, W., Israel, M., Evans, A. C., Karama, S., & for the Brain Development Cooperative Group. (2013). Testosterone-related cortical maturation across childhood and adolescence. Cerebral Cortex, 23(6), 1424–1432. 10.1093/cercor/bhs125 22617851 PMC3643718

[b62] Peng, H., Gong, W., Beckmann, C. F., Vedaldi, A., & Smith, S. M. (2021). Accurate brain age prediction with lightweight deep neural networks. Medical Image Analysis, 68, 101871. 10.1016/j.media.2020.101871 33197716 PMC7610710

[b63] Petersen, A. C., Crockett, L., Richards, M., & Boxer, A. (1988). A self-report measure of pubertal status: Reliability, validity, and initial norms. Journal of Youth and Adolescence, 17(2), 117–133. 10.1007/BF01537962 24277579

[b66] Raznahan, A., Shaw, P., Lalonde, F., Stockman, M., Wallace, G. L., Greenstein, D., Clasen, L., Gogtay, N., & Giedd, J. N. (2011). How does your cortex grow? Journal of Neuroscience, 31(19), 7174–7177. 10.1523/JNEUROSCI.0054-11.2011 21562281 PMC3157294

[b68] Schnack, H. G., van Haren, N. E. M., Nieuwenhuis, M., Hulshoff Pol, H. E., Cahn, W., & Kahn, R. S. (2016). Accelerated brain aging in schizophrenia: A longitudinal pattern recognition study. The American Journal of Psychiatry, 173(6), 607–616. 10.1176/appi.ajp.2015.15070922 26917166

[b70] Shaw, P., Gogtay, N., & Rapoport, J. (2010). Childhood psychiatric disorders as anomalies in neurodevelopmental trajectories. Human Brain Mapping, 31(6), 917–925. 10.1002/hbm.21028 20496382 PMC6870870

[b72] Smith, S. M., Vidaurre, D., Alfaro-Almagro, F., Nichols, T. E., & Miller, K. L. (2019). Estimation of brain age delta from brain imaging. NeuroImage, 200, 528–539. 10.1016/j.neuroimage.2019.06.017 31201988 PMC6711452

[b73] Somerville, L. H. (2016). Searching for signatures of brain maturity: What are we searching for? Neuron, 92(6), 1164–1167. 10.1016/j.neuron.2016.10.059 28009272

[b76] Tamnes, C. K., Walhovd, K. B., Grydeland, H., Holland, D., Østby, Y., Dale, A. M., & Fjell, A. M. (2013). Longitudinal working memory development is related to structural maturation of frontal and parietal cortices. Journal of Cognitive Neuroscience, 25(10), 1611–1623. 10.1162/jocn_a_00434 23767921

[b77] Tanner, J. M. (1962). Growth at adolescence, 2nd ed. Springfield.

[b78] Terry, M. B., Goldberg, M., Schechter, S., Houghton, L. C., White, M. L., O’Toole, K., Chung, W. K., Daly, M. B., Keegan, T. H. M., Andrulis, I. L., Bradbury, A. R., Schwartz, L., Knight, J. A., John, E. M., & Buys, S. S. (2016). Comparison of clinical, maternal, and self pubertal assessments: Implications for health studies. Pediatrics, 138(1), e20154571. 10.1542/peds.2015-4571 27279647 PMC4925080

[b79] Tervo-Clemmens, B., Calabro, F. J., Parr, A. C., Fedor, J., Foran, W., & Luna, B. (2022). A canonical trajectory of executive function maturation during the transition from adolescence to adulthood. PsyArXiv. 10.31234/osf.io/73yfv PMC10616171 37903830

[b82] Vidal-Pineiro, D., Wang, Y., Krogsrud, S. K., Amlien, I. K., Baaré, W. F., Bartres-Faz, D., Bertram, L., Brandmaier, A. M., Drevon, C. A., Düzel, S., Ebmeier, K., Henson, R. N., Junqué, C., Kievit, R. A., Kühn, S., Leonardsen, E., Lindenberger, U., Madsen, K. S., Magnussen, F.,… Fjell, A. (2021). Individual variations in ‘brain age’ relate to early-life factors more than to longitudinal brain change. ELife, 10, e69995. 10.7554/eLife.69995 34756163 PMC8580481

[b83] Vijayakumar, N., Op de Macks, Z., Shirtcliff, E. A., & Pfeifer, J. H. (2018). Puberty and the human brain: Insights into adolescent development. Neuroscience & Biobehavioral Reviews, 92, 417–436. 10.1016/j.neubiorev.2018.06.004 29972766 PMC6234123

[b84] Vijayakumar, N., Youssef, G. J., Allen, N. B., Anderson, V., Efron, D., Hazell, P., Mundy, L., Nicholson, J. M., Patton, G., Seal, M. L., Simmons, J. G., Whittle, S., & Silk, T. (2021). A longitudinal analysis of puberty‐related cortical development. NeuroImage, 228, 117684. 10.1016/j.neuroimage.2020.117684 33385548

[b85] Wierenga, L., Langen, M., Ambrosino, S., van Dijk, S., Oranje, B., & Durston, S. (2014). Typical development of basal ganglia, hippocampus, amygdala and cerebellum from age 7 to 24. NeuroImage, 96, 67–72. 10.1016/j.neuroimage.2014.03.072 24705201

[b86] Wierenga, L. M., Bos, M. G. N., Schreuders, E., Vd Kamp, F., Peper, J. S., Tamnes, C. K., & Crone, E. A. (2018). Unraveling age, puberty and testosterone effects on subcortical brain development across adolescence. Psychoneuroendocrinology, 91, 105–114. 10.1016/j.psyneuen.2018.02.034 29547741

