# Supplementary material for: BrainAGE as a measure of maturation during early adolescence
Source: Imaging Neurosci (Camb). 2023 Nov 30;1:imag-1-00037. doi: 10.1162/imag_a_00037 (PMC12007541; doi:10.1162/imag_a_00037)
Supplement: Supplementary Material [file imag_a_00037-supp.pdf]

Supplementary Materials

Supplementary Table 1. Brain features

	Volume	Area	Model 1 Training<br>(Not Included in Prediction)
Cortical	caudal anterior cingulate<br>caudal middle frontal<br>cuneus<br>entorhinal<br>fusiform<br>inferior parietal<br>inferior temporal<br>isthmus cingulate<br>lateral occipital<br>lateral orbitofrontal<br>lingual<br>medial orbitofrontal<br>middle temporal<br>parahippocampal<br>paracentral<br>pars opercularis<br>pars orbitalis<br>pars triangularis<br>pericalcarine<br>postcentral<br>posterior cingulate<br>precentral<br>precuneus<br>rostral anterior cingulate<br>rostral middle frontal<br>superior frontal<br>superior parietal<br>superior temporal<br>supramarginal<br>transverse temporal<br>left insula<br>left bankssts<br>frontal pole<br>temporal pole<br>Total GM	caudal anterior cingulate<br>caudal middle frontal<br>cuneus<br>entorhinal<br>fusiform<br>inferior parietal<br>inferior temporal<br>isthmus cingulate<br>lateral occipital<br>lateral orbitofrontal<br>lingual<br>medial orbitofrontal<br>middle temporal<br>parahippocampal<br>paracentral<br>pars opercularis<br>pars orbitalis<br>pars triangularis<br>pericalcarine<br>postcentral<br>posterior cingulate<br>precentral<br>precuneus<br>rostral anterior cingulate<br>rostral middle frontal<br>superior frontal<br>superior parietal<br>superior temporal<br>supramarginal<br>transverse temporal<br>left insula<br>left bankssts<br>frontal pole<br>temporal pole	BrainSeg_Vol<br>BrainSeg_Vol_No_Vent<br>BrainSeg_Vol_No_Vent_Surf<br>Total GM<br>SupraTentorial_Vol_No_Vent<br>SupraTentorial_No_Vent_Voxel_Count<br>Mask<br>BrainSegVol_eTIV_Ratio<br>MaskVol_eTIV_Ratio<br>Vessel<br>Choroid Plexus<br>Optic Chiasm
Subcortical	Thalamus<br>Caudate		

	Putamen<br>Pallidum<br>Lateral Ventricle<br>Inferior Lateral Ventricle<br>Hippocampus<br>Amygdala<br>Accumbens<br>Left Ventral DC<br>3rd Ventricle<br>4th Ventricle<br>BrainStem<br>Posterior CC<br>MidPosterior<br>Central CC<br>MidAnterior CC<br>Anterior CC<br>Total GM		
Other	Cerebellum WM<br>Cerebellum<br>Intercranial<br>CSF<br>SupraTentorial		

Supplementary Table 2. Reliability Measures

	Youth-Report PDS		Parent-Report PDS	
	Female	Male	Female	Male
Baseline	.60	.53	.70	.64
Follow-Up	.76	.73	.81	.79

Measure reliability, Cronbach's a as calculated in psych::omega()

Supplementary Table 3. Model 1 – Baseline, Youth-Report PDS
Regression results using corrected_gap as the criterion

Predictor	*b*	*b* 95% CI	*beta*	*beta* 95% CI	sr^2	sr^2 95% CI	*r*	Fit

		[LL, UL]		[LL, UL]		[LL, UL]		
(Intercept)	4.23**	[3.51, 4.95]						
youth_mean	0.25**	[0.16, 0.35]	0.07	[0.04, 0.09]	.00	[.00, .01]	.05**	
truth	-0.40**	[-0.47, -0.33]	-0.14	[-0.17, -0.12]	.02	[.01, .03]	-.14**	
								R^2 = .023** 95% CI[.02,.03]

Note. A significant *b*-weight indicates the beta-weight and semi-partial correlation are also significant. *b* represents unstandardized regression weights. *beta* indicates the standardized regression weights. sr^2 represents the semi-partial correlation squared. *r* represents the zero-order correlation. *LL* and *UL* indicate the lower and upper limits of a confidence interval, respectively. * indicates $p < .05$. ** indicates $p < .01$.

Supplementary Table 4. Model 1 – Baseline, Parent-Report PDS
Regression results using corrected_gap as the criterion

Predictor	*b*	*b* 95% CI [LL, UL]	*beta*	*beta* 95% CI [LL, UL]	sr^2	sr^2 95% CI [LL, UL]	*r*	Fit
(Intercept)	4.23**	[3.71, 4.76]						
parent_mean	0.24**	[0.17, 0.31]	0.07	[0.05, 0.09]	.00	[.00, .01]	.04**	
truth	-0.40**	[-0.45, -0.34]	-0.14	[-0.16, -0.12]	.02	[.01, .02]	-.13**	
								R^2 = .021** 95% CI[.02,.03]

Supplementary Table 5. Model 1 – Baseline, Cognition
Regression results using corrected_gap as the criterion

Predictor	*b*	*b* 95% CI [LL, UL]	*beta*	*beta* 95% CI [LL, UL]	sr^2	sr^2 95% CI [LL, UL]	*r*	Fit
(Intercept)	4.41**	[3.88, 4.95]						
nihtbx_totalcomp_uncorrected	-0.00	[-0.01, 0.00]	-0.02	[-0.04, 0.00]	.00	[-.00, .00]	-.06**	

truth	-0.35**	[-0.40, -0.29]	-0.12	[-0.14, -0.10]	.01	[.01, .02]	-.13**	
								R^2 = .017** 95% CI[.01,.02]

Supplementary Table 6. Model 1 – Follow-Up, Youth-Report PDS
Regression results using corrected_gap as the criterion

Predictor	*b*	*b* 95% CI [LL, UL]	*beta*	*beta* 95% CI [LL, UL]	sr^2	sr^2 95% CI [LL, UL]	*r*	Fit
(Intercept)	2.09**	[1.24, 2.93]						
youth_mean	0.50**	[0.42, 0.57]	0.16	[0.14, 0.19]	.02	[.02, .03]	.14**	
truth	-0.25**	[-0.32, -0.17]	-0.08	[-0.10, -0.06]	.01	[.00, .01]	-.03**	
								R^2 = .025** 95% CI[.02,.03]

Supplementary Table 7. Model 1 – Follow-Up, Parent-Report PDS
Regression results using corrected_gap as the criterion

Predictor	*b*	*b* 95% CI [LL, UL]	*beta*	*beta* 95% CI [LL, UL]	sr^2	sr^2 95% CI [LL, UL]	*r*	Fit
(Intercept)	2.37**	[1.54, 3.21]						
parent_mean	0.58**	[0.51, 0.65]	0.21	[0.18, 0.23]	.04	[.03, .05]	.18**	
truth	-0.29**	[-0.36, -0.21]	-0.09	[-0.12, -0.07]	.01	[.00, .01]	-.03**	
								R^2 = .040** 95% CI[.03,.05]

Supplementary Table 8. Model 2 – Baseline, Youth-Report PDS
Regression results using corrected_gap as the criterion

Predictor	*b*	*b* 95% CI [LL, UL]	*beta*	*beta* 95% CI [LL, UL]	sr^2	sr^2 95% CI [LL, UL]	*r*	Fit
(Intercept)	1.84**	[0.55, 3.13]						
youth_mea	0.31**	[0.14, 0.48]	0.07	[0.03,	.00	[-.00, .01]	.06**	

n				0.10]				
truth	-0.24**	[-0.37, -0.11]	-0.07	[-0.11, -0.03]	.00	[-.00, .01]	-.06**	
								R^2 = .008** 95% CI[.00,.02]

Supplementary Table 9. Model 2 – Baseline, Parent-Report PDS
Regression results using corrected_gap as the criterion

Predictor	*b*	*b* 95% CI [LL, UL]	*beta*	*beta* 95% CI [LL, UL]	sr^2	sr^2 95% CI [LL, UL]	*r*	Fit
(Intercept)	1.35**	[0.40, 2.30]						
parent_mean	0.19**	[0.06, 0.31]	0.04	[0.01, 0.07]	.00	[-.00, .00]	.03*	
truth	-0.17**	[-0.27, -0.08]	-0.05	[-0.08, -0.02]	.00	[-.00, .00]	-.04**	
								R^2 = .003** 95% CI[.00,.01]

Supplementary Table 10. Model 2 – Baseline, Cognition
Regression results using corrected_gap as the criterion

Predictor	*b*	*b* 95% CI [LL, UL]	*beta*	*beta* 95% CI [LL, UL]	sr^2	sr^2 95% CI [LL, UL]	*r*	Fit
(Intercept)	1.32**	[0.36, 2.29]						
nihtbx_totalcomp_uncorrected	0.00	[-0.00, 0.01]	0.01	[-0.02, 0.04]	.00	[-.00, .00]	-.00	
truth	-0.16**	[-0.26, -0.06]	-0.05	[-0.07, -0.02]	.00	[-.00, .00]	-.04**	
								R^2 = .002** 95% CI[.00,.00]

Supplementary Table 11. Model 2 – Follow-Up, Youth-Report PDS
Regression results using corrected_gap as the criterion

Predictor	*b*	*b* 95% CI [LL, UL]	*beta*	*beta* 95% CI [LL,	sr^2	sr^2 95% CI [LL, UL]	*r*	Fit

				UL]				
(Intercept)	-1.73	[-3.48, 0.03]						
youth_mean	0.40**	[0.25, 0.55]	0.09	[0.06, 0.13]	.01	[.00, .01]	.10**	
truth	0.08	[-0.08, 0.23]	0.02	[-0.02, 0.05]	.00	[-.00, .00]	.05**	
								R^2 = .010** 95% CI[.00,.02]

Supplementary Table 12. Model 2 – Baseline, Parent-Report PDS
Regression results using corrected_gap as the criterion

Predictor	*b*	*b* 95% CI [LL, UL]	*beta*	*beta* 95% CI [LL, UL]	sr^2	sr^2 95% CI [LL, UL]	*r*	Fit
(Intercept)	-1.43	[-3.18, 0.32]						
parent_mean	0.43**	[0.29, 0.56]	0.11	[0.07, 0.14]	.01	[.00, .02]	.11**	
truth	0.05	[-0.11, 0.20]	0.01	[-0.02, 0.05]	.00	[-.00, .00]	.05**	
								R^2 = .013** 95% CI[.01,.02]

Supplementary Figure 1. Overlaid model predictions

A

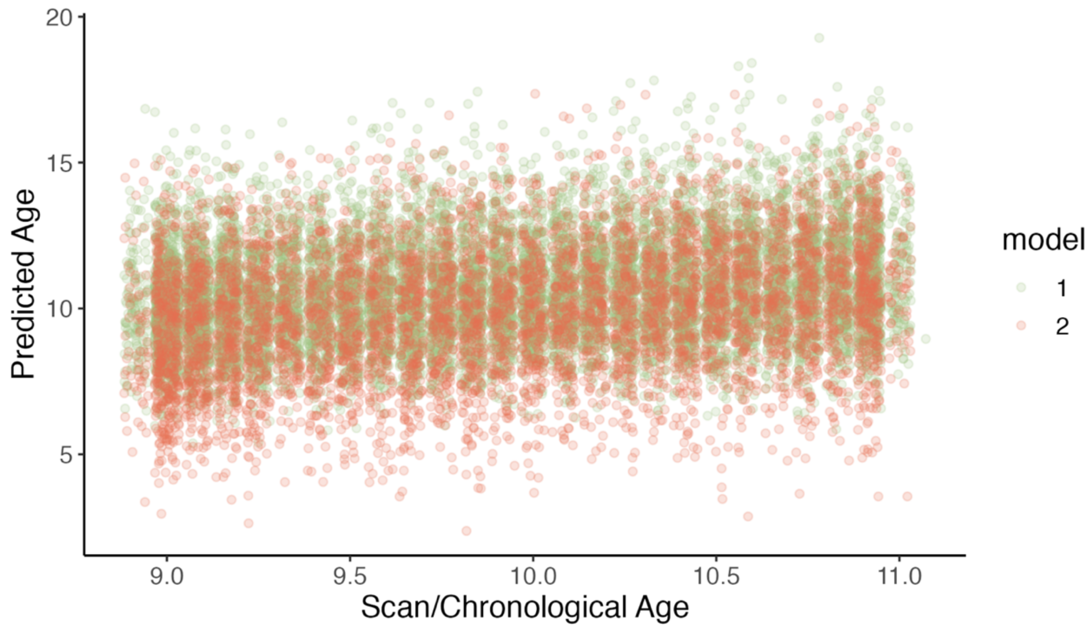

B

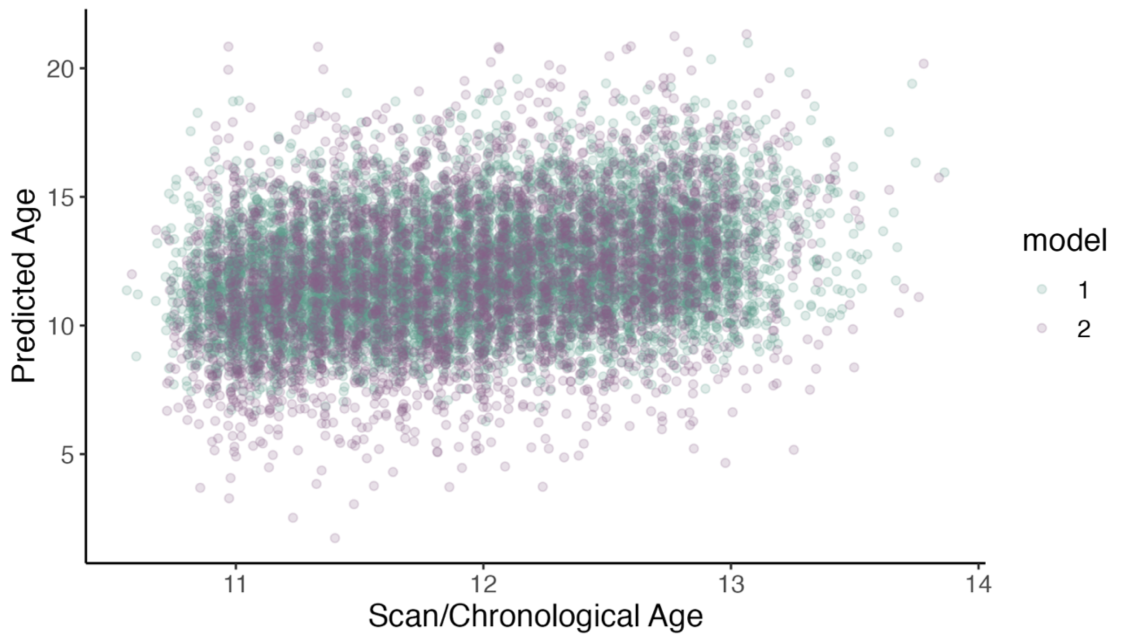

Figure S1. Overlaid model predictions. A) Both baseline models plotted on the same figure. B). Both follow-up models plotted on the same figure.
